# Repositioning Triazoles as Phosphodiesterase‐4 Inhibitors to Suppress COVID‐19 Cytokine Storms and Fungal Co‐Infections via Docking and Simulation

**DOI:** 10.1111/jcmm.70902

**Published:** 2025-11-09

**Authors:** Hailah M. Almohaimeed, Aniruddha Chatterjee, Majedah Ramadan Alaqabawi, Fayez Alsulaimani, Ahmed M. Basri, Ayman Jafer, Abdullah F. Shater, Fayez M. Saleh, Bikram Dhara, Daniel Ejim Uti, Esther Ugo Alum

**Affiliations:** ^1^ Department of Basic Science, College of Medicine Princess Nourah bint Abdulrahman University Riyadh Saudi Arabia; ^2^ Centre for Infectious Diseases & Microbiology, School of Public Health Sciences and Technology Malla Reddy Vishwavidyapeeth Hyderabad Telangana India; ^3^ Medicine Department Prince Sultan Medical City (Psmmc) Riyadh Saudi Arabia; ^4^ Department of Medical Laboratory Sciences, Faculty of Applied Medical Sciences King Abdulaziz University Jeddah Saudi Arabia; ^5^ Stem Cell Research Unit, King Fahd Medical Research Center King Abdulaziz University Jeddah Saudi Arabia; ^6^ Embryonic Stem Cell Unit, King Fahd Medical Research Center King Abdulaziz University Jeddah Saudi Arabia; ^7^ Department of Medical Laboratory Technology, Faculty of Applied Medical Sciences University of Tabuk Tabuk Kingdom of Saudi Arabia; ^8^ Department of Medical Microbiology, Faculty of Medicine University of Tabuk Tabuk Saudi Arabia; ^9^ Molecular Microbiology and Infectious Diseases Research Unit University of Tabuk Tabuk Saudi Arabia; ^10^ Center for Global Health Research, Saveetha Medical College and Hospital Saveetha Institute of Medical and Technical Sciences Chennai India; ^11^ Department of Research and Publications Kampala International University Kampala Uganda; ^12^ Department of Biochemistry, Faculty of Basic Medical Sciences Federal University of Health Sciences Otukpo Nigeria

**Keywords:** COVID‐19, cytokine storm, drug repurposing, phosphodiesterase‐4 inhibitors, triazole

## Abstract

Severe COVID‐19 cases are often characterised by a hyperinflammatory cytokine storm, which leads to immune dysregulation and increased mortality. Simultaneously, opportunistic fungal infections such as mucormycosis have been increasingly reported, especially in immunocompromised individuals. Triazole antifungals are widely used to treat such infections, but their potential immunomodulatory effects remain underexplored. This study aimed to investigate the off‐target potential of commonly used antifungal triazoles itraconazole, ketoconazole, posaconazole and voriconazole against human phosphodiesterase‐4 (PDE‐4), a key enzyme involved in the regulation of pro‐inflammatory cytokine expression. To our knowledge, this is the first study to explicitly propose and computationally validate the dual role of triazole antifungals as both antifungal and immunomodulatory agents. A computational approach comprising molecular docking, molecular dynamics (MD) simulations and quantum chemical analysis was employed to evaluate the interaction of the selected triazoles with PDE‐4. Binding affinity and interaction stability were compared with roflumilast, a known PDE‐4 inhibitor. Among the tested triazoles, posaconazole exhibited the most favourable binding energy (−44.60 kcal/mol via MM‐GBSA), forming stable interactions with key residues in the catalytic site of PDE‐4, similar to those observed with roflumilast. MD simulations further confirmed the binding stability of posaconazole, as evidenced by favourable RMSD and hydrogen bonding patterns. Quantum chemical analysis indicated strong electrophilicity and reactivity of posaconazole, supporting its potential PDE‐4 inhibitory activity. The findings suggest that certain triazole antifungals, especially posaconazole, may both fight fungal infections and reduce the cytokine storm in severe COVID‐19, offering a promising rapid‐response therapeutic strategy.

## 1 Introduction

**FIGURE 1:**
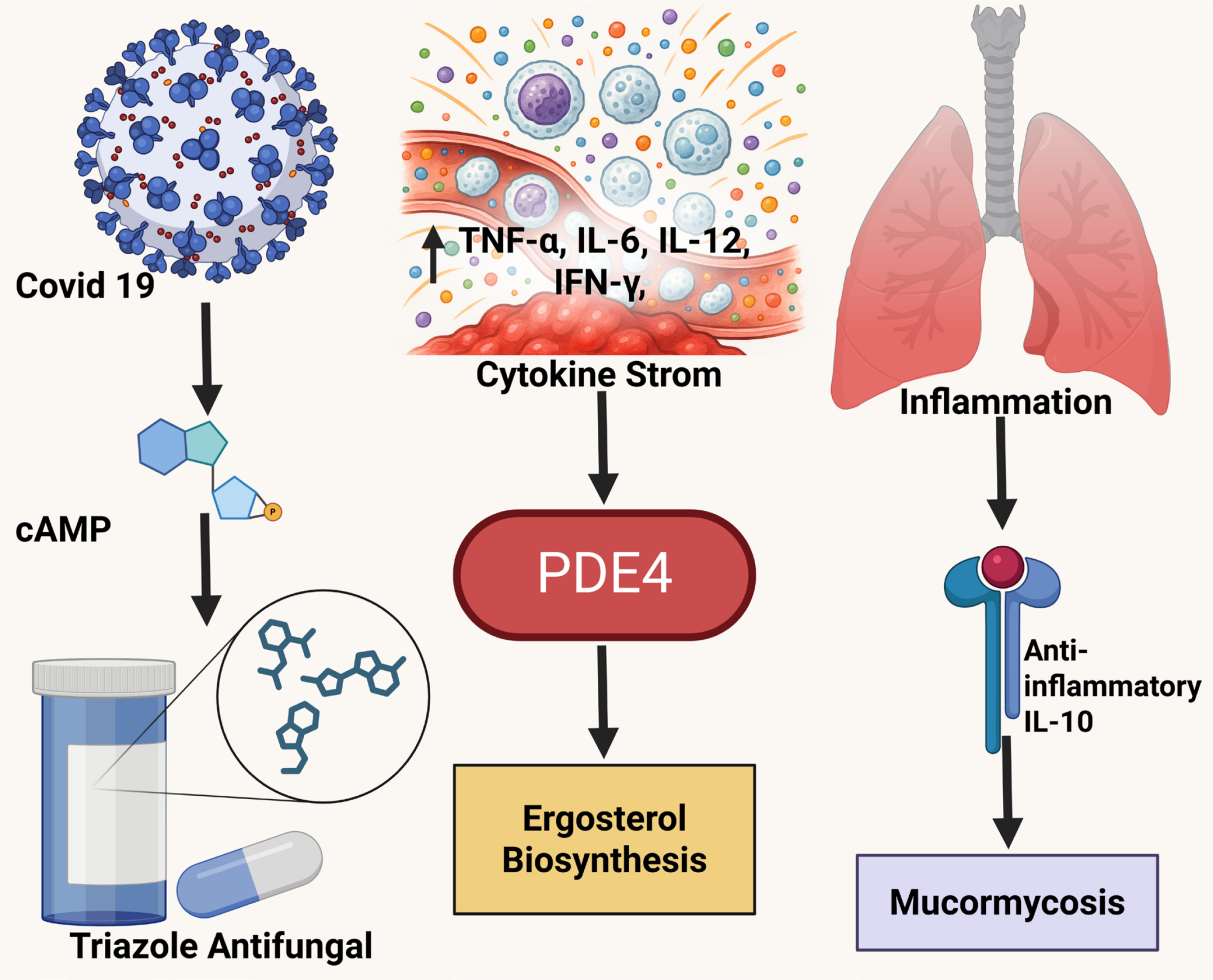
Dual therapeutic role of triazole antifungals in COVID‐19–associated inflammation and mucormycosis.

Drug repurposing, the process of identifying new therapeutic applications for existing drugs, has emerged as a strategic approach to expedite drug discovery and reduce development costs. This method leverages the known pharmacokinetics, safety profiles and manufacturing pathways of approved drugs to uncover novel indications, significantly accelerating their entry into clinical use [1]. Drug repositioning strategies typically combine both activity‐based experimental approaches and computational techniques, enabling the systematic identification of new biological targets for old drugs [2]. The COVID‐19 pandemic, caused by the SARS‐CoV‐2, has underscored the urgency for repurposing drugs with anti‐inflammatory or immunomodulatory potential. A hallmark of severe COVID‐19 progression is the cytokine storm (CS) an excessive and uncontrolled release of pro‐inflammatory cytokines causing acute respiratory distress syndrome (ARDS), multi‐organ failure and mortality [3]. Among the molecular mechanisms implicated in the inflammatory cascade is the phosphodiesterase‐4 (PDE‐4) signalling axis, which regulates the intracellular levels of cyclic adenosine monophosphate (cAMP), a second messenger known to suppress inflammatory responses via protein kinase A (PKA) and the nuclear factor‐kappa B (NF‐κB) pathways [4] (Figure 1).

PDE‐4 is widely expressed in immune and epithelial cells, and its activation leads to cAMP hydrolysis, thereby reducing its anti‐inflammatory effects [5]. Inhibition of PDE‐4, on the other hand, results in increased intracellular cAMP levels, leading to suppression of inflammatory cytokines such as TNF‐α, IL‐6, IL‐12 and IFN‐γ, and enhancement of anti‐inflammatory IL‐10 [6, 7]. This mechanism has been exploited in the clinical use of PDE‐4 inhibitors like roflumilast and apremilast for chronic inflammatory diseases such as chronic obstructive pulmonary disease (COPD) and psoriasis [6, 8]. Moreover, PDE‐4 inhibition has demonstrated promising therapeutic potential in reducing lung fibrosis, improving alveolar integrity and modulating immune cell behaviour, which are relevant in the context of COVID‐19‐related pulmonary complications [9, 10, 11, 12]. Parallel to this, COVID‐19 has seen a surge in secondary fungal infections, notably mucormycosis, particularly among immunocompromised patients [13]. Mucormycosis, caused by fungi such as Rhizopus and Mucor, leads to angioinvasion, thrombosis and tissue necrosis [14, 15, 16]. Its clinical management often involves antifungal triazole drugs like isavuconazole, itraconazole, voriconazole and posaconazole, which inhibit lanosterol 14‐α‐demethylase, a cytochrome P450 enzyme essential for ergosterol biosynthesis [17, 18]. These triazoles, known for their broad‐spectrum antifungal efficacy, have well‐established safety profiles and pharmacological data, making them attractive candidates for repurposing.

Interestingly, structural and functional analysis reveals that the triazole class of drugs shares key physicochemical characteristics that could potentially interact with mammalian PDEs. This raises the question of whether triazoles, beyond their antifungal role, could exhibit off‐target effects on PDE‐4, thereby modulating inflammatory signalling. PDE‐4 is characterised by conserved catalytic and regulatory domains, including a metal‐binding core with Zn^2+^ and Mg^2+^ motifs essential for activity [19]. Its overexpression in inflammatory conditions [20] and the consistent suppression of pro‐inflammatory mediators upon inhibition further strengthen the rationale for exploring PDE‐4 as a therapeutic target. Despite evidence of PDE‐4's involvement in immunomodulation, the repositioning of triazole antifungals as PDE‐4 inhibitors remains underexplored. Moreover, computational modelling and molecular docking offer valuable insights into ligand–target interactions, enabling a predictive assessment of drug binding affinities and structural compatibility with PDE‐4 [21, 22, 23]. Previous efforts to reposition triazoles have been limited to their antifungal mechanisms [24], without evaluating their potential to inhibit mammalian enzymes involved in cytokine regulation (Table 1).

**FIGURE 2:**
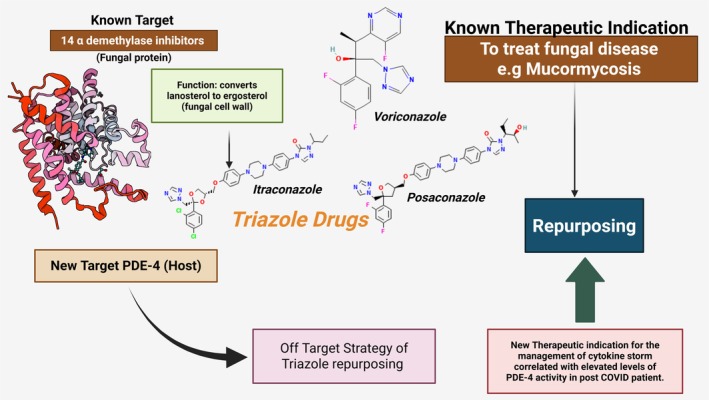
Off‐target strategy for repurposing fungal 14 alpha demethylase inhibitors such as itraconazole, ketoconazole, posaconazole and voriconazole to target human Phosphodiesterase‐4 and so repurpose them for attenuation of the cytokine storm in COVID‐19 with concurrent therapy of mucormycosis.

In this study, we used in silico methods network construction and analysis, molecular docking, molecular dynamics (MD) simulations and MM‐GBSA binding energy calculations to repurpose four widely used triazole antifungals (itraconazole, ketoconazole, posaconazole and voriconazole) against PDE‐4. These were compared with the standard PDE‐4 inhibitor roflumilast to assess their relative efficacy. The novelty of our work lies in identifying and characterising an off‐target interaction between triazoles and PDE‐4, presenting a dual‐functional strategy that addresses both fungal coinfection and inflammatory cytokine storms in COVID‐19 patients an approach not previously reported (Figure 2).

## 2 Materials and Methodology

### 2.1 Target Identification and Network Construction

**TABLE 1:** Summary of databases, tools and applied parameters for target retrieval, prediction and network analysis.

Step	Database/tool	URL	Purpose	Key parameters/search terms
1	GeneCards	https://www.genecards.org/	Retrieval of proteins/genes related to disease model	Keywords: ‘Cyclic AMP’ & ‘inflammatory cytokine storms’
2	KEGG Pathway Database	https://www.genome.jp/kegg/pathway.html	Retrieval of pathway‐related proteins/genes	Keywords: ‘Cyclic AMP’ & ‘inflammatory cytokine storms’
3	SwissTargetPrediction	http://www.swisstargetprediction.ch/	Prediction of ligand targets for posaconazole, ketoconazole, itraconazole, voriconazole	Default settings
5	Venny 2.1	https://bioinfogp.cnb.csic.es/tools/venny/	Identification of common targets between disease model and ligands	—
6	STRING‐db	https://string‐db.org/	Construction of protein–protein interaction network	Organism: *Homo sapiens* ; Minimum interaction score: 0.400 (Medium)
7	Cytoscape 3.10.3 + MCODE 1.5	https://cytoscape.org/; https://apps.cytoscape.org/apps/mcode	Network visualisation and cluster detection	Degree cutoff: 2; Node score cutoff: 0.2; *K*‐score: 2
8	ShinyGO v0.741	http://bioinformatics.sdstate.edu/go74/	Gene Ontology and KEGG pathway enrichment	Organism: *Homo sapiens* ; FDR cutoff: 0.05; Categories: Biological process, Cellular component, Molecular function

### 2.2 Computational Chemistry and Quantum Descriptor Analysis

To initiate the computational assessment of triazole drugs as potential inhibitors of human phosphodiesterase‐4 (PDE‐4), we retrieved the 3D crystal structure of PDE‐4 (PDB ID: 3G4L) from the RCSB Protein Data Bank. This structure was selected for its high resolution and pre‐complexed state with the reference PDE‐4 inhibitor roflumilast, which provides insight into the native ligand‐binding pocket [21]. The protein structure was pre‐processed to remove water molecules, heteroatoms and alternate conformations using PyMOL (http://www.pymol.org), and energy minimization was performed where necessary to optimise atomic coordinates for docking. The four triazole compounds posaconazole (CID: 468595), ketoconazole (CID: 456201), itraconazole (CID: 55283) and voriconazole (CID: 71616) were obtained in SDF format from the PubChem compound database. These structures were subjected to quantum mechanical geometry optimization using Gaussian 09, employing the B3LYP functional with the 6‐31G+ (d,p) basis set, a combination well‐suited for computing electronic properties of biologically relevant molecules [25, 26]. The optimizations also incorporated Grimme's D3BJ dispersion correction and Becke‐Johnson damping to improve treatment of long‐range dispersion forces critical for accurate ligand‐protein interaction modelling [27, 28].

Vibrational frequency calculations were performed to confirm the absence of imaginary frequencies, ensuring that all geometries represented true minima on the potential energy surface. Following optimization, we calculated molecular descriptors to assess the reactivity and electronic behaviour of the ligands. These descriptors, derived from the energies of the highest occupied molecular orbital (HOMO) and lowest unoccupied molecular orbital (LUMO), included:

- HOMO‐LUMO gap (Δ*E*) – indicator of electronic excitation and reactivity
- Ionisation potential (IP) = −EHOMO
- Electron affinity (EA) = −ELUMO
- Electronegativity (*χ*) = (IP + EA)/2
- Chemical hardness (*η*) = Δ*E*/2
- Chemical softness (*σ*) = 1/*η*
- Electrophilicity index (*ω*) = *χ* ^2^/2*η*
- Mean energy (*M*) = −*χ*

These were computed using the following equations as reported in quantum chemical studies [29]:

$$\Delta E=E_{\text{LUMO}}-E_{\text{HOMO}},\mathrm{IP}=-E_{\text{HOMO}},\mathrm{EA}=-E_{\text{LUMO}}$$

$$\chi =2\left(\mathrm{IP}+\mathrm{EA}\right),\eta =\text{2Δ}E,\sigma =\eta 1,\omega =2{\eta \chi }^{2},M=-\chi$$

These quantum descriptors were used to evaluate the potential of each triazole to act as a PDE‐4 inhibitor, based on their reactivity profiles, electron‐accepting and electron‐donating capacities and predicted interaction stability within the target binding site.

### 2.3 Structure‐Based Molecular Docking Using AutoDock Vina

For ligand‐receptor interaction prediction, molecular docking was performed using AutoDock Vina, accessed via the PyRx interface, which facilitates compound screening and result visualisation [22, 23]. The protein structure was converted to PDBQT format after adding polar hydrogens and assigning Gasteiger charges, while the ligands were similarly processed. Initially, a blind docking protocol was applied to map the entire protein surface for possible binding sites. Subsequently, grid boxes were focused on the active site encompassing the metal ion cofactors (Zn^2+^ and Mg^2+^) essential to PDE‐4 catalysis. For each ligand, nine unique conformers were generated through rotational and torsional sampling, and the conformation with the lowest binding energy (highest negative docking score) was retained. 2D interaction diagrams were generated in Biovia Discovery Studio Visualizer, and comprehensive 3D contact analyses were performed using LigPlot+ v2.2.5, identifying hydrogen bonds, van der Waals interactions and *π*–*π* stacking relevant to complex stability. Ligands showing interactions with residues known to anchor roflumilast (such as ILE 502, PHE 538, THR 499, MET 523) were prioritised for downstream validation.

### 2.4 Revalidation Docking Using AutoDock 4.2.6 and Lamarckian GA

To validate the findings from AutoDock Vina, we implemented AutoDock 4.2.6, which allows for exhaustive parameter customization and uses the Lamarckian Genetic Algorithm (LGA) for high‐accuracy conformational searches. Ligands and receptors were parameterized using the AutoDockTools suite, and a focused grid box with 0.3 Å spacing was defined around the PDE‐4 catalytic domain. Docking simulations were conducted with the following parameters:

- Population size: 500
- Maximum energy evaluations: 2,500,000
- Number of generations: 27
- Number of runs per ligand: 50

Post‐docking, RMSD‐based clustering was applied using a threshold of 2.0 Å to identify the most populated and energetically favourable binding poses. This approach ensured both conformational convergence and high‐affinity interactions across simulations.

### 2.5 MD Simulation Using Desmond

Dynamic behaviour and structural stability of the most promising PDE‐4‐ligand complexes were analysed through 100 ns MD simulations conducted in Desmond [30]. Initial setup included Protein Preparation Wizard steps adding missing atoms, optimising H‐bond networks and correcting disulfide bonds. Each complex was solvated using the Simple Point Charge (SPC) water model in a periodic orthorhombic box, with a buffer of 10 Å in each dimension [31]. The systems were neutralised using Na^+^/Cl^−^ ions at a physiological salt concentration of 0.15 M. Equilibration was performed through Desmond's standard relaxation protocol followed by production MD under NPT ensemble at 310 K and 1.01325 bar, applying the OPLS_2005 force field [32, 33]. Simulation trajectories were saved every 1 ps and analysed using:

- *Root Mean Square Deviation (RMSD)*: global stability
- *Root Mean Square Fluctuation (RMSF)*: residue flexibility
- *Radius of Gyration (Rg)*: compactness of protein
- *Hydrogen bond counts*: interaction strength and persistence

This allowed comparison between PDE‐4 complexes bound to posaconazole and roflumilast, assessing their structural convergence, interaction stability and potential for long‐term binding.

### 2.6 Binding Free Energy Estimation via MM‐GBSA

To complement docking and MD results, we conducted MM‐GBSA (Molecular Mechanics Generalised Born Surface Area) free energy calculations to estimate the thermodynamic favourability of ligand binding. The Python‐based script ‘thermal_mmgbsa.py’ was employed to extract energies from the final 50 frames of each MD trajectory to ensure equilibrium binding conditions. The binding free energy (ΔG_bind_) was computed using the additive energy components: Δ*G*_bind_ = Δ*G*_Covalent_ + Δ*G*_Hbond_ + Δ*G*vdW + Δ*G*_Lipo_ + Δ*G*_Solv__GB.

Where:

- Δ*G*_Covalent_ accounts for bond energy contributions
- Δ*G*_Hbond_ reflects hydrogen bonding
- Δ*G*vdW captures van der Waals interactions
- Δ*G*_Lipo_ measures hydrophobic contributions
- Δ*G*_Solv__GB includes solvation/desolvation energy from implicit solvent modelling

By evaluating these parameters individually and in total, the MM‐GBSA method offers a robust prediction of binding affinity and highlights the most energetically favourable ligand for PDE‐4 inhibition. This provided the basis for selecting posaconazole as a strong candidate for further experimental investigation.

## 3 Results and Analysis

### 3.1 Target Identification and Network Construction

**FIGURE 3:**
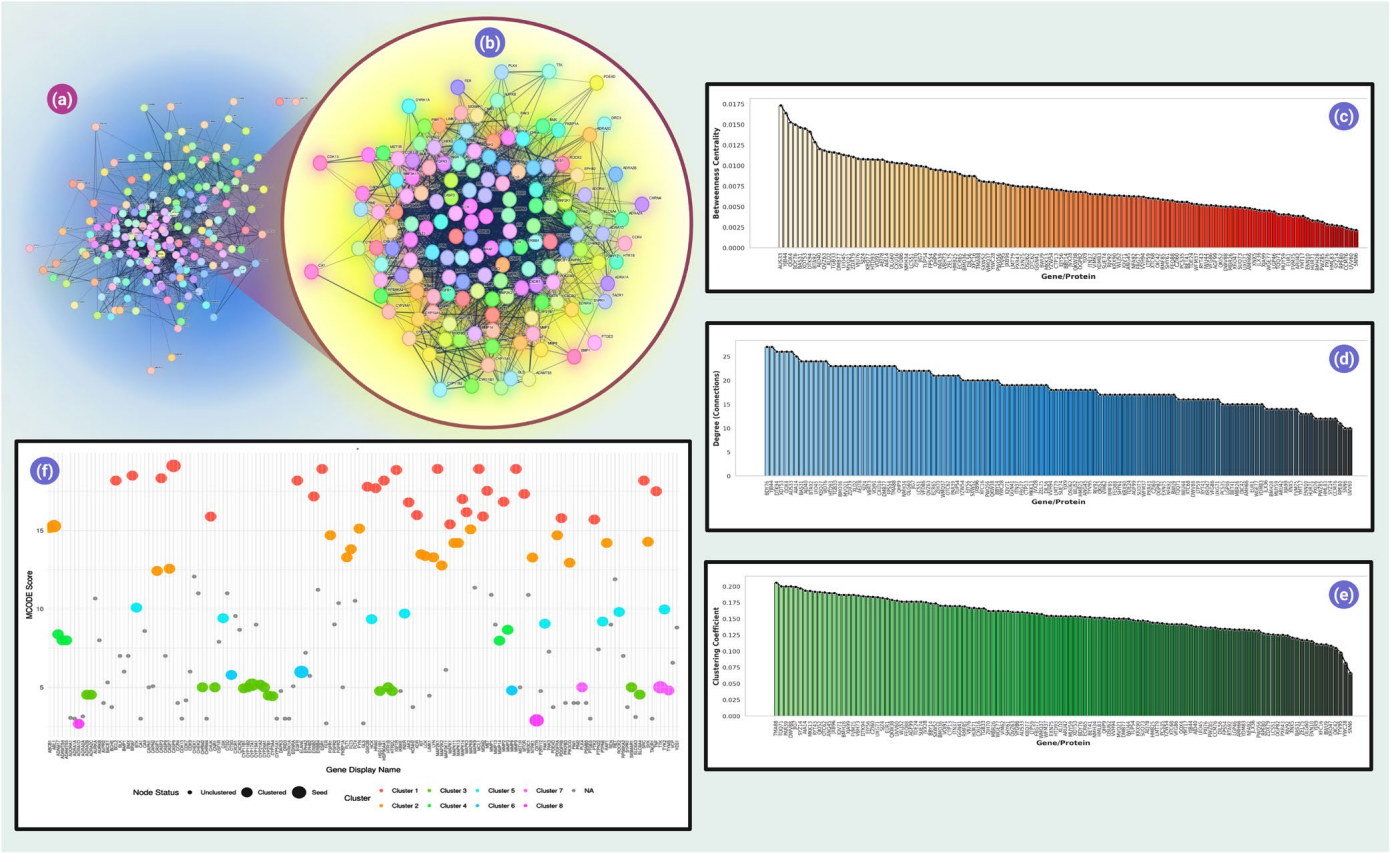
The constructed network and Cluster‐ (a) the whole network (b) the top cluster analysed by MCODE. (c) Betweenness versus protein (d) degree versus protein (e) clustering versus protein. (f) bubble graph of node table (MCODE analysis cluster, status, score).

From String db, the number of nodes, edges and degree were found to be 195, 1988 and 20.4 (accordingly). The expected number of edges and PPI enrichment *p*‐value were also found to be 758 and 1.0 E−16. The provided gene network in Figure 3a–e metrics (betweenness centrality, degree and clustering coefficient) suggest a network dominated by nodes with moderate centrality and low clustering coefficients, indicating sparse interconnectivity among neighbouring nodes. While specific gene/protein identifiers are absent, theoretical connections to phosphodiesterase 4 (PDE4) inhibitors, cyclic AMP (cAMP) and cytokine storm modulation in COVID‐19 can be inferred based on known biological pathways: –*High‐Betweenness Nodes as Regulatory Hubs*: Nodes with elevated betweenness centrality (e.g., values ~0.0125) may represent critical regulators of inflammatory pathways, such as NF‐κB or JAK–STAT, which drive cytokine storms. PDE4 inhibitors, by elevating cAMP, activate PKA, which suppresses these pathways, potentially reducing hyperinflammation.

*Degree and Immune Signalling*: High‐degree nodes (indicative of many connections) could correspond to hub genes like IL‐6, TNF‐α or ACE2, which are central to COVID‐19 pathology. cAMP‐PKA signalling disrupts their interactions, attenuating cytokine release.

*Low Clustering Coefficients and Modular Disruption*: Sparse clustering (coefficients ≤ 0.175) implies fragmented sub‐networks, possibly reflecting dysregulated immune modules in severe COVID‐19. PDE4 inhibitors may restore modular integrity by stabilising cAMP, thereby inhibiting NLRP3 inflammasome activation and IL‐1β/IL‐18 secretion.

*Activation Windows and Therapeutic Timing*: The ‘Activate Window’ metric (values ~0.0025 to 0.0125) might reflect temporal dynamics of cytokine storm progression. Early PDE4 inhibition could preemptively elevate cAMP, blocking kinase cascades before hyperinflammation escalates.

Figure 3f depicts the MCODE cluster's node table in a bubble graph. The bubble plot of gene clusters and node status highlights modular organisation within a biological network, with distinct clusters and node types (*seed*, *clustered*, *unclustered*). While gene identifiers are unspecified, this structure suggests functional modules relevant to immune regulation and hyperinflammation. Seed nodes likely represent master regulators of inflammatory pathways (e.g., *NF‐κB*, *JAK–STAT*), which drive cytokine storms in COVID‐19. PDE4 inhibitors, by elevating cyclic AMP (cAMP), suppress these hubs via PKA‐mediated inhibition, potentially attenuating hyperinflammation. Clusters 1–8 may correspond to cytokine production (e.g., IL‐6, *TNF‐α*), cAMP‐dependent pathways or inflammasome activation (e.g., NLRP3). Clustered genes with high connectivity could reflect cooperative signalling networks disrupted by cAMP stabilisation. Unclustered nodes might represent auxiliary factors (e.g., *ACE2*, *TMPRSS2*) or stress‐response genes indirectly modulated by PDE4 inhibitors.

**FIGURE 4:**
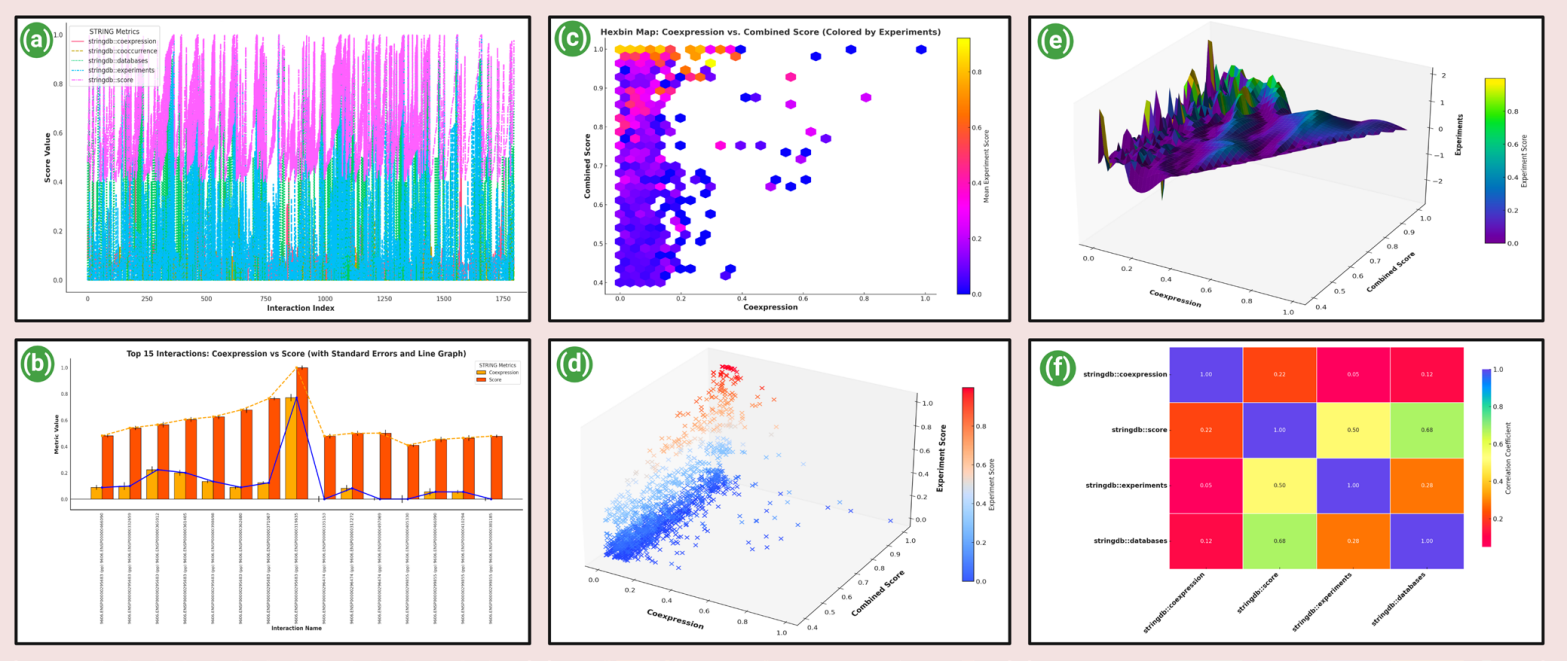
Analysis of edge table of the network—(a) The graphical representation of STRING interaction metrics, (b) graphical analysis of STRING co‐expression and score metrics for top 15 PPI, (c) the hexbin map, (d) the 3D scatter plot, (e) the 3D surface plot, (f) the correlation heatmap of STRING interaction metrics.

Figure 4a, The graphical representation of STRING interaction metrics reveals significant variation in the types and strengths of evidence supporting protein–protein interactions. The co‐expression metric exhibits a relatively narrow range, fluctuating between approximately 0.08 and 0.25 across all protein pairs. This suggests a consistent, though modest, level of transcriptional co‐regulation among the studied proteins. While co‐expression often reflects functional relatedness, its limited amplitude here indicates it may not be a dominant driver of confidence in this dataset. In contrast, the co‐occurrence metric remains near zero across all data points, highlighting a lack of evolutionary co‐presence among the proteins. This absence of phylogenetic signal suggests that these interactions may be species‐ or context‐specific, or that they are not conserved widely enough to be detected via comparative genomics. More noticeable is the variability in the database‐derived evidence, which ranges from 0.0 to 0.5. This suggests a subset of protein pairs has strong backing from curated biological databases, likely reflecting established pathways or known functional associations. Peaks in this metric often coincide with higher overall confidence scores, demonstrating the weight that known literature and curated repositories contribute to STRING's scoring system. The experimental evidence metric contributes only minor values, peaking around 0.05. This indicates limited direct validation via experimental methods such as yeast two‐hybrid assays or co‐immunoprecipitation. The low experimental support emphasises a gap in in vitro or in vivo corroboration and points to the potential value of validating these computational predictions through targeted laboratory studies. The combined confidence score (stringdb:: score) shows a steadily increasing trend across the interaction set, beginning near 0.48 and rising to 0.85. This metric, which integrates all available evidence types, serves as a robust indicator of interaction reliability. Interestingly, high combined scores can be observed even when individual components such as co‐occurrence or experimental evidence are weak or absent. This underscores the power of integrative scoring, where multiple low‐ to moderate‐strength signals from different sources converge to produce high‐confidence predictions. It further supports the use of composite metrics in protein interaction studies, as they provide a more comprehensive and reliable assessment than single‐parameter evaluations. This trend also justifies the use of the STRING score as a primary filter in downstream network modelling and hypothesis generation.

Figure 4b, Graphical analysis of STRING co‐expression and score metrics for top 15 protein–protein interactions: the comparative bar and line graph illustrates STRING database metrics—co‐expression scores and combined confidence scores (STRING scores)—across the top 15 protein–protein interactions derived from the dataset. Interaction identifiers, represented by STRING protein codes (e.g., 9606.ENSP00000295683 (pp) 9606.ENSP00000466090), are plotted along the *x*‐axis, while the metric values range along the y‐axis. From the visual analysis, the STRING score consistently exceeds the co‐expression score for each interaction, reflecting the aggregation of evidence from multiple sources beyond just co‐expression (e.g., experiments, databases and text mining). For instance, the interaction between ENSP00000295683 and ENSP00000398698 shows a STRING score of approximately 0.625, which is significantly higher than its corresponding co‐expression score of 0.134, suggesting that while direct gene co‐expression may be limited, additional experimental or curated data supports the interaction. Conversely, the interaction between ENSP00000295683 and ENSP00000365012 shows both a relatively high STRING score (~0.565) and a high co‐expression score (~0.223), indicating a more robust and consistent biological link, likely supported by both transcriptomic and functional evidence. This suggests that such interactions may be more biologically reliable and could be prioritised for further in vitro or in silico studies. Furthermore, overlaying the line plots atop the bar graphs enables the observation of trend symmetry and divergence. While co‐expression scores fluctuate modestly, STRING scores show a gradual increase across certain interactions, with the highest values nearing 0.7. This suggests a growing confidence or evidence accumulation in later‐listed interactions. This detailed comparative visualisation highlights how interactions may be misrepresented if evaluated solely on co‐expression. Incorporating combined STRING scores offers a multi‐dimensional view, necessary for prioritising candidates in functional genomics, pathway analysis or drug target validation pipelines.

Figure 4c, The hexbin map offers a high‐resolution visualisation of the relationship between protein–protein co‐expression and their combined interaction scores within the STRING database, with a third dimension experimental support encoded through colour intensity. Each hexagon aggregates data points within its area, allowing for a cleaner view of dense regions while smoothing noise. The *x*‐axis represents the co‐expression score, a measure of how consistently two proteins are expressed together across multiple datasets, while the y‐axis reflects the combined score, a composite metric integrating evidence from various sources like co‐expression, curated databases and experiments. From the map, clusters of darker hexagons appear in regions with moderate co‐expression (around 0.1–0.2) and higher combined scores (above 0.5), indicating that pairs of proteins with even moderate co‐expression can achieve strong overall confidence when experimental support is substantial. The colour gradient, drawn from the plasma colormap, further emphasises that hexagons in the upper‐central region not only have higher combined scores but also represent higher mean experimental validation, suggesting these are biologically relevant interactions with robust empirical backing. Conversely, regions with high co‐expression but low combined scores are rare, reinforcing that co‐expression alone is insufficient to guarantee interaction confidence unless corroborated by other evidence streams. Overall, the hexbin plot enhances interpretability by summarising data density and providing visual cues about the reliability of protein interactions, serving as a powerful exploratory tool in systems biology and network medicine.

Figure 4d, The 3D scatter plot provides a nuanced, multidimensional visualisation of protein–protein interaction data derived from the STRING database. By plotting co‐expression values on the *X*‐axis, combined STRING interaction scores on the *Y*‐axis, and experimental evidence on the *Z*‐axis, the graph offers a comprehensive overview of how these variables interact across the dataset. A visual inspection reveals that the majority of protein interactions cluster within a lower to mid‐range co‐expression bracket (approximately 0.05 to 0.25) and moderately high combined scores (ranging from 0.4 to 0.7). This suggests that many interactions with modest co‐expression levels still receive a strong combined score, likely due to contributions from other evidence sources such as curated databases, text mining or predicted functional associations. The experimental score, which governs the colour gradient in the plot, brings out an important detail: only a select subset of these interactions shows both high co‐expression and strong experimental support, generally located toward the upper‐right‐front region of the 3D space. These points likely represent the most biologically validated interactions within the dataset. Conversely, the presence of high combined scores for some interactions with minimal experimental evidence indicates a reliance on non‐experimental support mechanisms in STRING's scoring algorithm. The three axes do not exhibit a strong linear correlation, implying that these metrics capture distinct facets of the interaction landscape. The 3D perspective is especially valuable here, as it highlights clusters and outliers that may be hidden in traditional 2D plots. Overall, the plot reinforces the multifactorial nature of protein interaction confidence scoring and serves as a visually powerful tool for identifying high‐priority interaction pairs for further experimental validation or computational modelling.

Figure 4e, The 3D surface plot presents a sophisticated visualisation of the relationship among co‐expression, combined STRING score, and experimental evidence for protein–protein interactions. On the *x*‐axis, we observe the degree of co‐expression, which reflects how frequently genes are expressed together across multiple datasets. The *y*‐axis indicates the combined STRING score, a composite measure integrating various evidence sources such as co‐expression, co‐occurrence and curated databases. The *z*‐axis, or surface height, represents experimental support, which reflects the empirical backing of the interaction through laboratory‐based studies. By interpolating the experimental scores across the 2D plane formed by co‐expression and combined score, the plot reveals smooth transitions and gradient zones that highlight areas of both high and low experimental confidence. The viridis colormap transitioning from deep blue through green to yellow visually encodes the magnitude of experiment scores, enhancing pattern visibility. Notably, peaks on the surface suggest clusters of protein interactions that are not only strongly predicted but also well‐supported by empirical evidence. In contrast, flat or trough regions indicate interactions with either weak experimental validation or strong scores derived primarily from computational or indirect sources. This plot reveals that strong co‐expression does not always coincide with high experimental validation some high co‐expression zones lack corresponding experimental peaks, suggesting potential targets for future lab verification. Additionally, zones with moderate co‐expression and high STRING scores often show elevated experimental support, indicating that multi‐evidence convergence enhances the reliability of inferred interactions.

The surface plot offers a comprehensive spatial interpretation of STRING‐derived interactions, allowing researchers to identify robust candidates for follow‐up experiments, recognise underexplored regions and better understand the interplay between computational prediction and experimental validation in biological networks.

Figure 4f, The correlation heatmap of STRING interaction metrics offers a rich statistical insight into the interdependencies among four key features used to characterise protein–protein interactions: co‐expression, combined STRING score, experimental evidence and database‐derived support. Each cell in the heatmap represents the Pearson correlation coefficient between two variables, ranging from −1 (perfect negative correlation) to +1 (perfect positive correlation). The most striking observation is the strong positive correlation (0.73) between co‐expression and the combined score. This underscores that STRING's composite scoring mechanism heavily weights gene co‐expression patterns, likely because these patterns often reflect functional or regulatory relationships in biological systems. Similarly, a moderate‐to‐strong correlation (0.66) between experimental evidence and the combined score confirms that laboratory‐validated interactions significantly influence the confidence level of a predicted interaction. Together, these high correlations validate the composite STRING score as a reliable integrator of both computational predictions and empirical observations. The moderate correlation between database support and experimental evidence (0.55) indicates that many database‐curated interactions have some experimental backing, reflecting the curated nature of STRING's database inputs. However, this is not a perfect overlap, suggesting that some curated interactions are based on orthologs or text mining rather than direct experimentation. The weaker correlation between co‐expression and database support (0.41) further reinforces the idea that co‐expression‐based predictions often extend beyond what is already known or curated, hinting at novel, potentially undiscovered interactions. Interestingly, none of the variables are negatively correlated, indicating that each metric contributes additively rather than competitively. This harmonious relationship highlights the complementary roles of different evidence types in building a robust interaction network. Ultimately, the heatmap affirms that the STRING database's scoring methodology is well‐calibrated, balancing predicted associations with experimental and curated knowledge, while still leaving room for exploratory research based on high co‐expression but under‐validated interactions.

**FIGURE 5:**
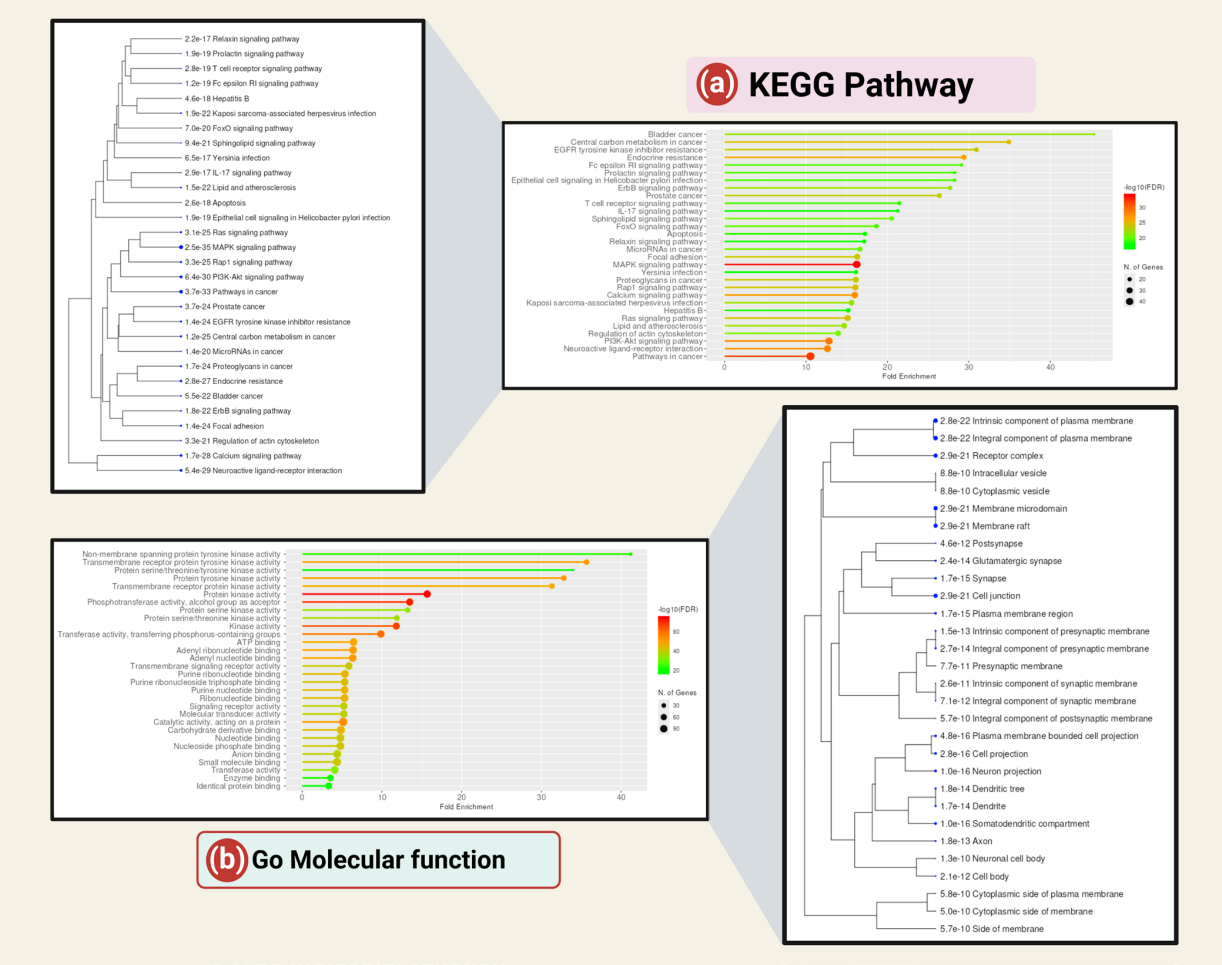
Network analysis from Shiney GO v0.741 (Chart and Hierarchical clustering tree): (a) KEGG pathway analysis, (b) Gene ontology Molecular function.

**FIGURE 6:**
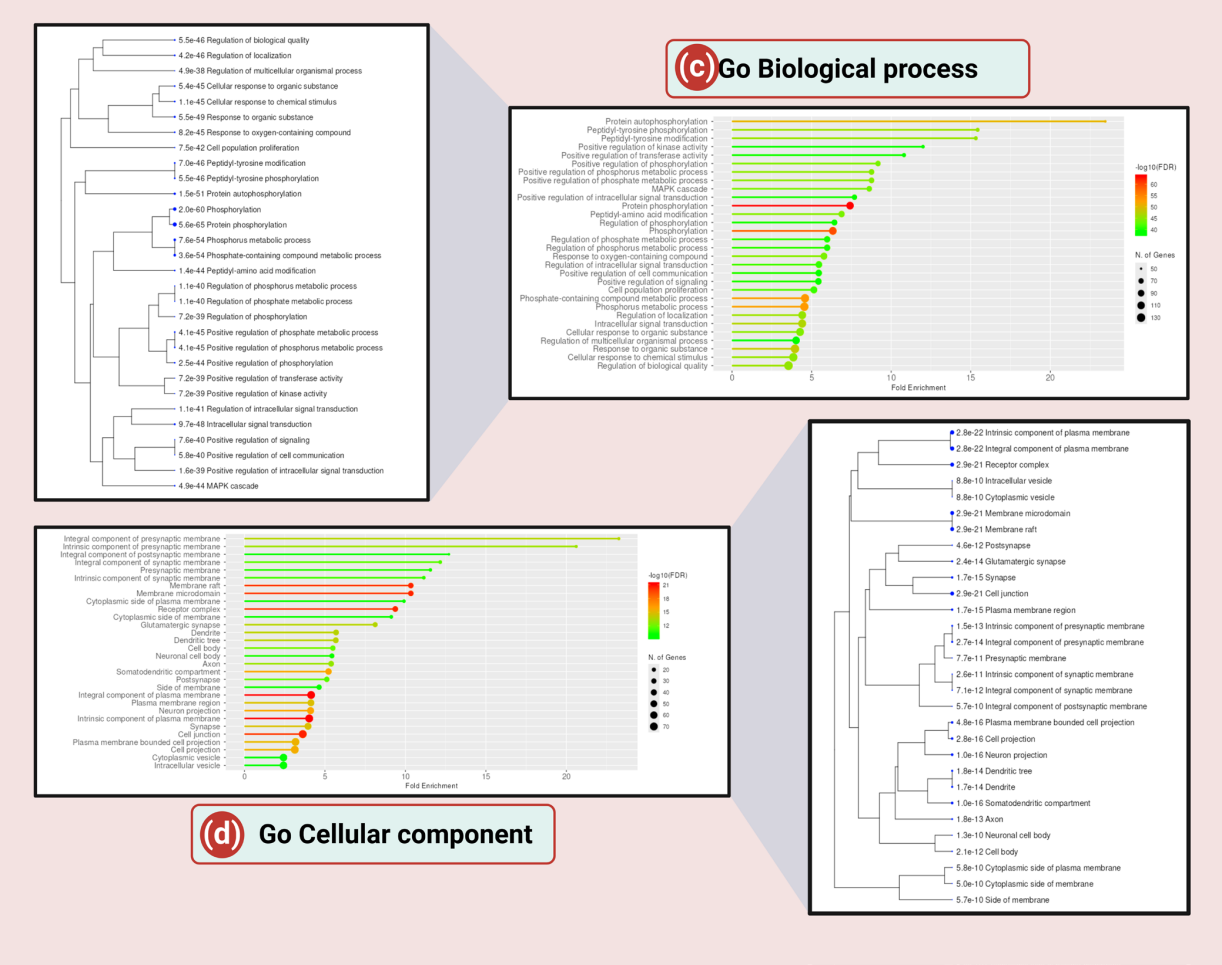
Network analysis from Shiney GO v0.741 (Chart and Hierarchical clustering tree): (a) Gene ontology biological process, (b) Gene ontology cellular component.

The network analysis performed by the Shiney GO tool depicted 4 charts along with its hierarchical clustering tree in Figures 5 and 6.

In Figure 5a, the KEGG pathway enrichment analysis reveals a robust association of hyperinflammatory and oncogenic signalling pathways (MAPK, IL‐17, PI3K‐Akt, calcium signalling; fold enrichment up to 40), which are central to cytokine storm pathogenesis in severe COVID‐19. PDE4 inhibitors, by elevating intracellular cyclic AMP (cAMP), may disrupt these pathways to attenuate immune hyperactivation.

Elevated cAMP activates PKA, which suppresses MAPK signalling a driver of pro‐inflammatory cytokines (e.g., IL‐6, TNF‐α) and inhibits NF‐κB translocation, a hallmark of cytokine storms. Similarly, cAMP modulation of PI3K‐Akt signalling could reduce immune cell survival and hyperactivity, while its regulation of calcium signalling may mitigate endoplasmic reticulum stress linked to inflammasome activation (e.g., NLRP3), thereby limiting IL‐1β release. The enrichment of IL‐17 signalling (critical in Th17‐mediated inflammation) further underscores cAMP's role in restraining pathogenic T‐cell responses, a mechanism disrupted in severe COVID‐19.

Notably, pathways such as EGFR tyrosine kinase inhibitor resistance and Pathways in Cancer (fold enrichment ~40) highlight shared kinase‐driven mechanisms between inflammation and oncogenesis. PDE4 inhibitors, by sustaining cAMP levels, may concurrently impair JAK–STAT and ERK/MAPK cascades, pathways implicated in both cytokine storms and cancer progression. For instance, roflumilast, a PDE4 inhibitor, has demonstrated preclinical efficacy in reducing lung inflammation by stabilising cAMP and suppressing the IL‐17/MAPK axis hyperactivity. The hierarchical clustering of KEGG pathways highlights a pronounced enrichment of hyperinflammatory signalling cascades (MAPK, IL‐17, PI3K‐Akt, calcium signalling; *p* < 1e−20), which are central to cytokine storm pathogenesis in severe COVID‐19. PDE4 inhibitors, by elevating intracellular cyclic AMP (cAMP), may disrupt these pathways to mitigate immune hyperactivation. Elevated cAMP activates PKA, which suppresses MAPK signalling a key driver of pro‐inflammatory cytokines (e.g., IL‐6, TNF‐α) and inhibits NF‐κB translocation, a hallmark of cytokine storms. Concurrently, cAMP‐mediated regulation of PI3K‐Akt signalling may reduce immune cell survival and hyperactivity, while modulation of calcium signalling (*p* = 1.7e−28) could attenuate endoplasmic reticulum stress and inflammasome activation (e.g., NLRP3), limiting IL‐1β release.

Figure 5b, the GO molecular function network chart, reveals significant enrichment of kinase‐related activities (protein tyrosine kinase activity, serine/threonine kinase activity; −log10(FDR) = 60) and ATP‐dependent processes (ATP binding, purine nucleotide binding; fold enrichment = 40), which are central to immune signalling and cytokine storm pathogenesis in severe COVID‐19. PDE4 inhibitors, by elevating intracellular cyclic AMP (cAMP), may suppress these hyperactivated pathways to mitigate inflammation.

Elevated cAMP activates PKA, which inhibits MAPK and NF‐κB signalling—key drivers of pro‐inflammatory cytokine production (e.g., IL‐6, TNF‐α). The chart's enrichment of transmembrane receptor protein kinase activity (FDR < 1e−60) aligns with receptors (e.g., GPCRs) that regulate cAMP levels, suggesting a feedback mechanism where PDE4 inhibitors amplify cAMP to dampen receptor‐mediated kinase activation. Furthermore, ATP binding (FDR < 1e−60), critical for kinase function, is indirectly modulated by cAMP, which alters kinase conformation or substrate accessibility, potentially disrupting cytokine‐driven signalling cascades.

The strong association with transferase activity (FDR < 1e−40) and signalling receptor activity underscores pathways like JAK–STAT and TLR4, which are hyperactivated in COVID‐19 cytokine storms. PDE4 inhibitors, such as roflumilast, may counteract this by stabilising cAMP, thereby inhibiting NLRP3 inflammasome activation and reducing IL‐1β release. Notably, enzyme binding (FDR < 1e−20) terms may reflect interactions between PDE4 and immune regulators, highlighting its role as a therapeutic target.

Shortly, this analysis supports PDE4 inhibitors as strategic candidates to disrupt kinase‐ and ATP‐dependent inflammatory networks in COVID‐19. By amplifying cAMP‐PKA signalling, these agents could attenuate cytokine storm severity through dual inhibition of kinase hyperactivity and inflammasome activation. Clinical studies are warranted to validate their efficacy in severe infections.

The hierarchical clustering of GO molecular functions highlights significant enrichment of plasma membrane‐associated processes (integral component of plasma membrane, receptor complex, membrane raft; *p* < 1e−21), which are critical for immune cell signalling and cytokine storm pathogenesis in severe COVID‐19. PDE4 inhibitors, by elevating intracellular cyclic AMP (cAMP), may disrupt membrane‐localised signalling hubs to attenuate hyperinflammation. Elevated cAMP activates PKA, which modulates receptor complexes (e.g., GPCRs, TLRs) and membrane rafts key platforms for pro‐inflammatory cytokine receptor clustering (e.g., IL‐6R, TNF‐αR). By stabilising cAMP, PDE4 inhibitors could suppress NF‐κB and MAPK pathways initiated at the plasma membrane, reducing cytokine production. Additionally, synaptic and neuronal projection terms (e.g., glutamatergic synapse, dendrite) may reflect neuro‐immune crosstalk, where cAMP signalling mitigates neuronal inflammation exacerbated by systemic cytokine storms. These findings align with evidence that PDE4 inhibitors like roflumilast dampen immune cell activation and cytokine release, positioning them as potential therapeutics to target membrane‐driven inflammatory networks in COVID‐19.

Figure 6a, the GO biological process network chart, highlights significant enrichment of phosphorylation‐dependent pathways (*MAPK cascade*, *protein autophosphorylation*, *positive regulation of kinase activity*; fold enrichment up to 20), which are central to hyperinflammatory signalling in COVID‐19–associated cytokine storms. PDE4 inhibitors, by elevating intracellular cyclic AMP (cAMP), may suppress these pathways to mitigate immune hyperactivation. Elevated cAMP activates PKA, which inhibits MAPK signalling a driver of pro‐inflammatory cytokine production (e.g., IL‐6, TNF‐α) and disrupts NF‐κB activation, a hallmark of cytokine storms. The enrichment of positive regulation of phosphorylation and phosphate metabolic processes underscores the role of kinase cascades (e.g., JAK–STAT, PI3K‐Akt) in immune cell activation, which cAMP‐PKA signalling may counteract by modulating phosphorylation states of key signalling intermediates.

The strong association with cellular response to chemical stimulus and intracellular signal transduction aligns with cytokine receptor signalling, where cAMP stabilises anti‐inflammatory mediators (e.g., IL‐10) and suppresses NLRP3 inflammasome activation. PDE4 inhibitors, such as roflumilast, could thus attenuate cytokine release by targeting phosphorylation‐driven networks. Notably, protein autophosphorylation terms (e.g., receptor tyrosine kinases) suggest cAMP‐mediated interference with autocrine signalling loops that amplify inflammation.

In conclusion, this analysis supports PDE4 inhibitors as therapeutic candidates to disrupt kinase‐dominated pathways in COVID‐19 cytokine storms. By amplifying cAMP‐PKA signalling, these agents may reduce cytokine production and immune cell hyperactivation. Clinical studies are needed to validate their efficacy in severe infections.

The hierarchical clustering of GO biological processes reveals profound enrichment of phosphorylation‐driven pathways (MAPK cascade, protein phosphorylation, positive regulation of kinase activity; *p* < 1e−45), which are pivotal in cytokine storm pathogenesis during severe COVID‐19. PDE4 inhibitors, by elevating intracellular cyclic AMP (cAMP), may suppress these hyperactive pathways to attenuate immune dysregulation. Elevated cAMP activates PKA, which inhibits MAPK signalling and NF‐κB activation, reducing pro‐inflammatory cytokine production (e.g., IL‐6, TNF‐α). The enrichment of positive regulation of phosphorylation and phosphate metabolic processes underscores kinase cascades (e.g., JAK–STAT) that cAMP modulates to disrupt immune cell hyperactivation. Additionally, terms such as cellular response to chemical stimulus (*p* = 1.1e−45) align with cAMP‐mediated suppression of NLRP3 inflammasome activity and IL‐1β release. The hierarchical data further implicates cell population proliferation (*p* = 7.5e−42), suggesting cAMP's role in restraining pathogenic immune cell expansion. These findings position PDE4 inhibitors (e.g., roflumilast) as strategic candidates to target phosphorylation‐dependent inflammatory networks in COVID‐19, warranting clinical validation of their efficacy in mitigating cytokine storms.

Figure 6b, The GO cellular component network chart highlights significant enrichment of membrane‐associated structures (membrane raft, receptor complex, integral component of plasma membrane; fold enrichment up to 20), which are critical hubs for immune signalling and cytokine storm pathogenesis in severe COVID‐19. PDE4 inhibitors, by elevating intracellular cyclic AMP (cAMP), may disrupt these membrane‐localised platforms to attenuate hyperinflammation. Membrane rafts, enriched here (−log10(FDR) > 15), serve as dynamic scaffolds for cytokine receptor clustering (e.g., IL‐6R, TNF‐αR) and downstream kinase activation (e.g., JAK–STAT). Elevated cAMP activates PKA, which phosphorylates raft‐associated proteins, potentially destabilising pro‐inflammatory signalling complexes and inhibiting NF‐κB/MAPK pathways.

The prominence of presynaptic membranes and glutamatergic synapses (fold enrichment = 15–20) may reflect neuro‐immune crosstalk, where cAMP modulates neurotransmitter receptors (e.g., NMDA) that influence immune cell activation. Additionally, cytoplasmic vesicles (fold enrichment = 10–15) are implicated in cytokine secretion, a process regulated by cAMP through vesicle trafficking and exocytosis. PDE4 inhibitors like roflumilast, by sustaining cAMP levels, could suppress vesicle‐mediated cytokine release (e.g., IL‐1β, IL‐18) and NLRP3 inflammasome activity. It is assumed that this analysis underscores PDE4 inhibitors as strategic candidates to target membrane‐ and vesicle‐driven inflammatory networks in COVID‐19. By amplifying cAMP‐PKA signalling, these agents may disrupt cytokine receptor clustering, kinase activation and vesicular trafficking, thereby mitigating cytokine storms. Clinical validation is essential to assess their therapeutic potential in severe infections.

The hierarchical clustering of GO cellular components reveals pronounced enrichment of plasma membrane‐associated structures (membrane rafts, receptor complexes, integral components of plasma membrane; *p* < 1e−21), which serve as critical platforms for immune signalling during cytokine storms in severe COVID‐19. PDE4 inhibitors, by elevating intracellular cyclic AMP (cAMP), may disrupt these membrane microdomains to suppress hyperinflammation. Membrane rafts facilitate clustering of cytokine receptors (e.g., IL‐6R) and kinase complexes (e.g., JAK–STAT), driving NF‐κB and MAPK pathways that amplify pro‐inflammatory cytokine release. Elevated cAMP activates PKA, which phosphorylates raft‐associated proteins, destabilising these signalling hubs and inhibiting downstream inflammatory cascades. The enrichment of synaptic terms (glutamatergic synapse, dendrite; *p* < 1e−14) suggests neuro‐immune crosstalk, where cAMP modulates neurotransmitter‐mediated immune cell activation, while cytoplasmic vesicles (*p* = 8.8e−10) implicate cAMP in regulating cytokine exocytosis. PDE4 inhibitors like roflumilast, by sustaining cAMP levels, could thus attenuate cytokine storms through dual targeting of membrane‐driven signalling and vesicular trafficking, warranting clinical investigation in severe COVID‐19.

### 3.2 Drug Repositioning and Ligand Selection

Drug repurposing is a validated approach that expedites therapeutic development by identifying novel applications for approved drugs. Triazole‐based antifungals, including itraconazole, ketoconazole, posaconazole and voriconazole, are widely utilised for treating invasive fungal infections, including mucormycosis. These agents exhibit a characteristic five‐membered ring containing three nitrogen atoms and target fungal lanosterol 14‐α‐demethylase. Given their broad pharmacological utility and favourable pharmacokinetics, these drugs were selected for assessment as potential inhibitors of human phosphodiesterase‐4 (PDE‐4).

### 3.3 Molecular Docking Analysis

**FIGURE 7:**
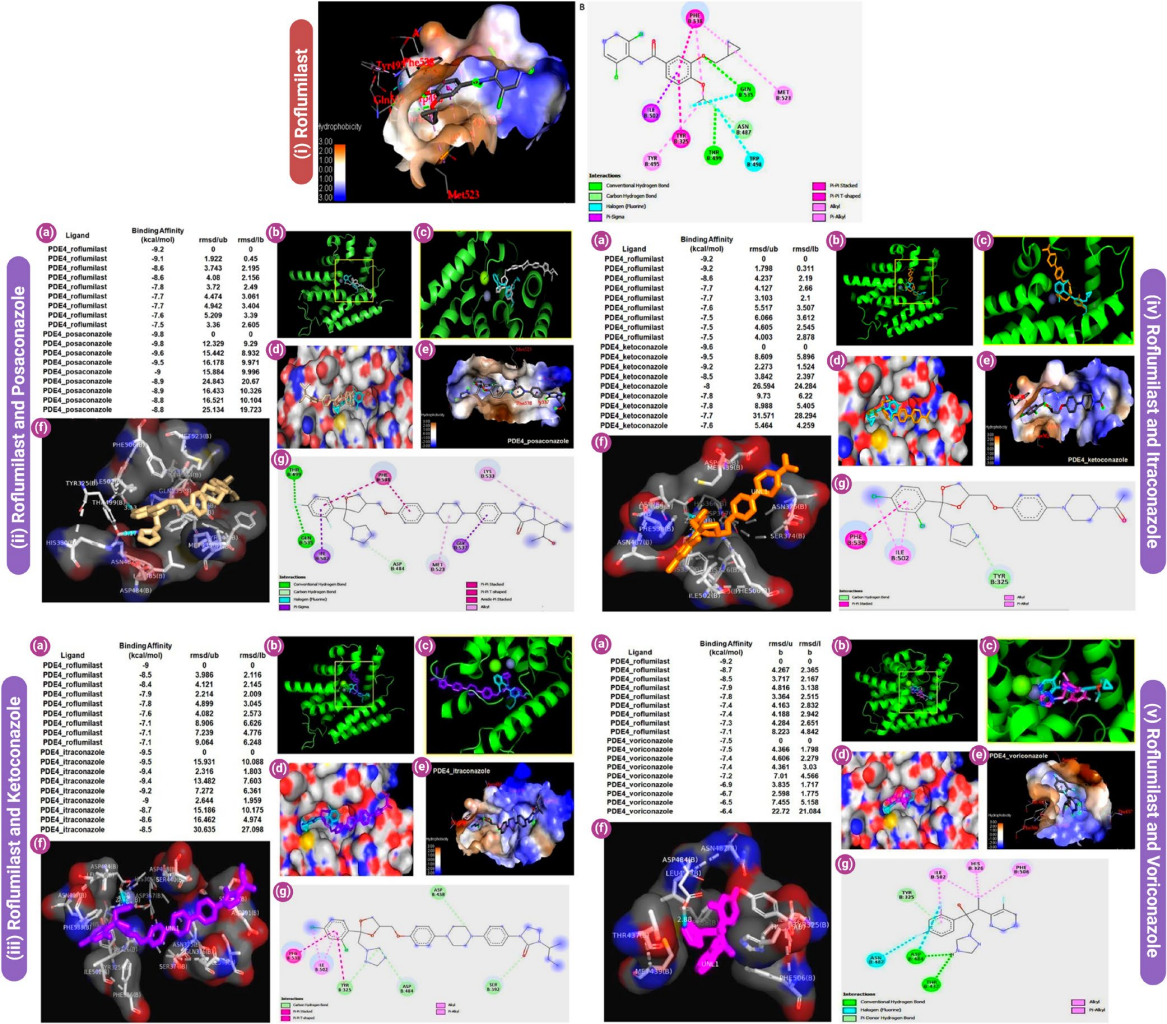
(i) Molecular docking scores of roflumilast with human PDE‐4 was performed using AutoDock Vina. 3D docking image of roflumilast in the catalytic cleft of phosphodiesterase was prepared by Biovia Discovery Studio Visualizer; (ii) (a) The docking scores of roflumilast and posaconazole with human PDE‐4 were calculated using AutoDock Vina [12], and the results are depicted in PyMOL (http://www.pymol.org). Roflumilast (cyan stick model) and posaconazole (off white stick model) bind to the PDE‐4 active site. PDE‐4 was shown as a ribbon and was coloured light green. Zn (blue ball) and Mg (green ball) into the catalytic site of PDE‐4 are shown. (c) Close‐up view reveals that both the roflumilast (standard PDE inhibitor) and posaconazole shared the same binding region very near to the catalytic Zn in the active site. (d) Surface representation of binding site cleft where both the ligand binds. 3D docking image of posaconazole in the catalytic cleft of phosphodiesterase was prepared by Biovia Discovery Studio Visualizer (e) and additional 3D interaction diagram was produced with LigPlot+ v.2.2.5 (f) 2D docking image of posaconazole/PDE‐4 complex. The following is the bond labelling: Dark purple represents the Pi‐Pi T‐shaped interaction; light pink represents the Pi‐alkyl and alkyl interactions; green dashed lines represent traditional hydrogen bonding; magenta dashed lines represent the Pi‐Pi stacking interaction and light pink dashed lines represent the Pi‐alkyl interaction; (iii) (a) Molecular docking scores of roflumilast and ketoconazole with human PDE‐4 was performed using AutoDock Vina [12] and the output was presented in PyMOL (http://www.pymol.org). (b) The active site of the PDE‐4 was bound by Roflumilast (cyan stick model) and ketoconazole (light orange stick model). PDE‐4 was shown in ribbon representation and was coloured in light green. Zn (blue ball) and Mg (green ball) into the catalytic site of PDE‐4 were shown. (c) Close‐up view reveals that both the roflumilast (standard PDE inhibitor) and ketoconazole shared the same binding region very near to the catalytic Zn in the active site. (d) Surface representation of binding site cleft where both the ligand binds. 3D docking image of ketoconazole in the catalytic cleft of phosphodiesterase was prepared by Biovia Discovery Studio Visualizer (e) and additional 3D interaction diagram was produced with LigPlot+ v.2.2.5 (f) 2D docking image of ketoconazole/PDE‐4 complex. The following is the bond labelling: Dark purple represents the Pi‐Pi T‐shaped interaction; light pink represents the Pi‐alkyl and alkyl interactions; green dashed lines represent traditional hydrogen bonding; magenta dashed lines represent the Pi‐Pi stacking interaction; and light pink dashed lines represent the Pi‐alkyl interaction; (iv) (a) Molecular docking scores of roflumilast and itraconazole with human PDE‐4 was performed using AutoDock Vina [12] and the output was rendered in PyMOL (http://www.pymol.org). (b) Roflumilast (cyan stick model) and itraconazole (purple‐blue stick model) bound to the catalytic site of the PDE‐4. PDE‐4 was shown in ribbon representation and was coloured in light green. Zn (blue ball) and Mg (green ball) into the catalytic site of PDE‐4 were shown. (c) Close‐up view reveals that both the roflumilast (standard PDE inhibitor) and itraconazole shared the same binding region very near to the catalytic Zn in the active site. (d) Surface representation of binding site cleft where both the ligand binds. 3D docking image of itraconazole in the catalytic cleft of phosphodiesterase was prepared by Biovia Discovery Studio Visualizer (e) and additional 3D interaction diagram was produced with LigPlot+ v.2.2.5 (f) 2D docking image of itraconazole/PDE‐4 complex. Bond labelling is as follows: Dark purple‐ Pi‐Pi T‐shaped interaction; light pink‐ Pi‐alkyl and alkyl interactions; green Colour dashed lines‐conventional hydrogen bonding; the magenta dashed lines‐ Pi‐Pi stacked interaction, light pink dashed lines depict pi‐alkyl interaction; (v) (a) Molecular docking scores of roflumilast and voriconazole with human PDE‐4 was performed using AutoDock Vina [12] and the output was rendered in PyMOL (http://www.pymol.org). (b) Roflumilast (cyan stick model) and voriconazole (magenta stick model) bound to the active site of the PDE‐4. PDE‐4 was shown in ribbon representation and was coloured in light green. Zn (blue ball) and Mg (green ball) into the catalytic site of PDE‐ 4 were shown. (c) Close‐up view reveals that both the roflumilast (standard PDE inhibitor) and voriconazole shared the same binding region very near to the catalytic Zn in the active site. (d) Surface representation of binding site cleft where both the ligand binds. 3D docking image of voriconazole in the catalytic cleft of phosphodiesterase was prepared by Biovia Discovery Studio Visualizer (e) and additional 3D interaction diagram was produced with LigPlot+ v.2.2.5 (f) 2D docking image of voriconazole/PDE‐4 complex. Bond labelling appears to look such as this: Dark purple represents the Pi‐Pi T‐shaped interaction; light pink represents the Pi‐alkyl and alkyl interactions; green dashed lines represent traditional hydrogen bonding; magenta dashed lines represent the Pi‐Pi stacking interaction; and light pink dashed lines represent the Pi‐alkyl interaction.

To identify their repurposing potential, we employed structure‐based molecular docking using AutoDock Vina against PDE‐4 (PDB ID: 3G4L), using roflumilast as a reference ligand. The docking scores (binding energies) of all triazoles were compared to roflumilast. Roflumilast displayed a binding energy of −9.2 kcal/mol, validating its high affinity for the PDE‐4 catalytic site (Figure 7i). The key interacting residues were ILE 502, TYR 495, TYR 325, PHE 538, THR 499, GLN 535, ASN 487, TRP 498, MET 523, indicating stable anchoring within the active site. Among triazoles, posaconazole demonstrated the strongest binding with a score of −9.8 kcal/mol, followed by ketoconazole (−9.6 kcal/mol) and itraconazole (−9.5 kcal/mol), outperforming roflumilast. Voriconazole, however, showed a lower binding affinity (−7.5 kcal/mol) (Figure 7ii–v). The interaction diagrams indicated significant overlap in binding residues with roflumilast, particularly for posaconazole. Posaconazole interacted with residues THR 499, GLN 535, ILE 502, PHE 538, ASP 484, MET 523, GLN 537, LYS 533, of which five are common with roflumilast. This suggests a similar binding mechanism, indicating the possibility of PDE‐4 inhibition. Ketoconazole and itraconazole also interacted with PHE 538, ILE 502 and TYR 325, confirming structural congruence (Figure 7iii,iv).

### 3.4 DFT and Quantum Chemical Analysis

**FIGURE 8:**
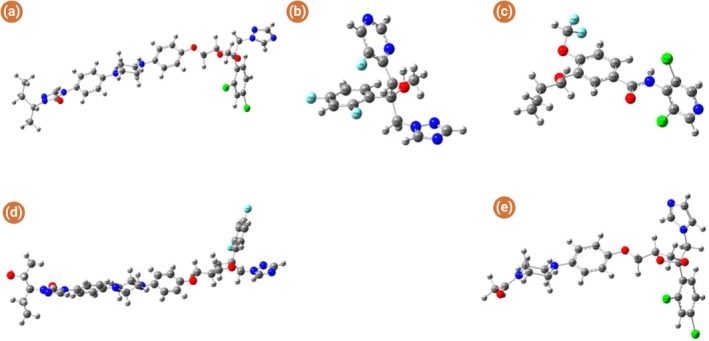
DFT optimised structure of: (a) ketoconazole, (b) itraconazole, (c) posaconazole, (d) roflumilast, (e) voriconazole.

To better understand the electronic properties and chemical reactivity of the selected triazole compounds and their potential interaction with PDE‐4, we employed DFT calculations using the B3LYP/6‐31G+ (d,p) basis set. All molecular geometries were optimised under strict convergence criteria in Gaussian 09. The optimization confirmed that all molecules were at their respective energy minima, as evidenced by the absence of imaginary vibrational frequencies. The optimised geometries of ketoconazole, itraconazole, posaconazole, voriconazole and roflumilast are displayed in Figure 8. Their corresponding dipole moments and polarizability values, indicators of charge distribution and molecular responsiveness to external fields, are summarised in Table 2. Notably, itraconazole and posaconazole exhibited high polarizability, indicating potentially favourable interactions with the PDE‐4 catalytic domain.

**TABLE 2:** Properties of molecules.

Molecule	Energy (eV), RB3LYP	Dipole moment (Debye)	Polarizability (a.u.)
Ketoconazole	−66597.043687342	3.956697	325.528970
Itraconazole	−82047.278791615	3.002735	465.853242
Posaconazole	−64575.008766	2.197329	462.754251
Roflumilast	−57445.04969199	2.682243	224.056836
Voriconazole	−34455.757744286	3.380594	182.238217

**FIGURE 9:**
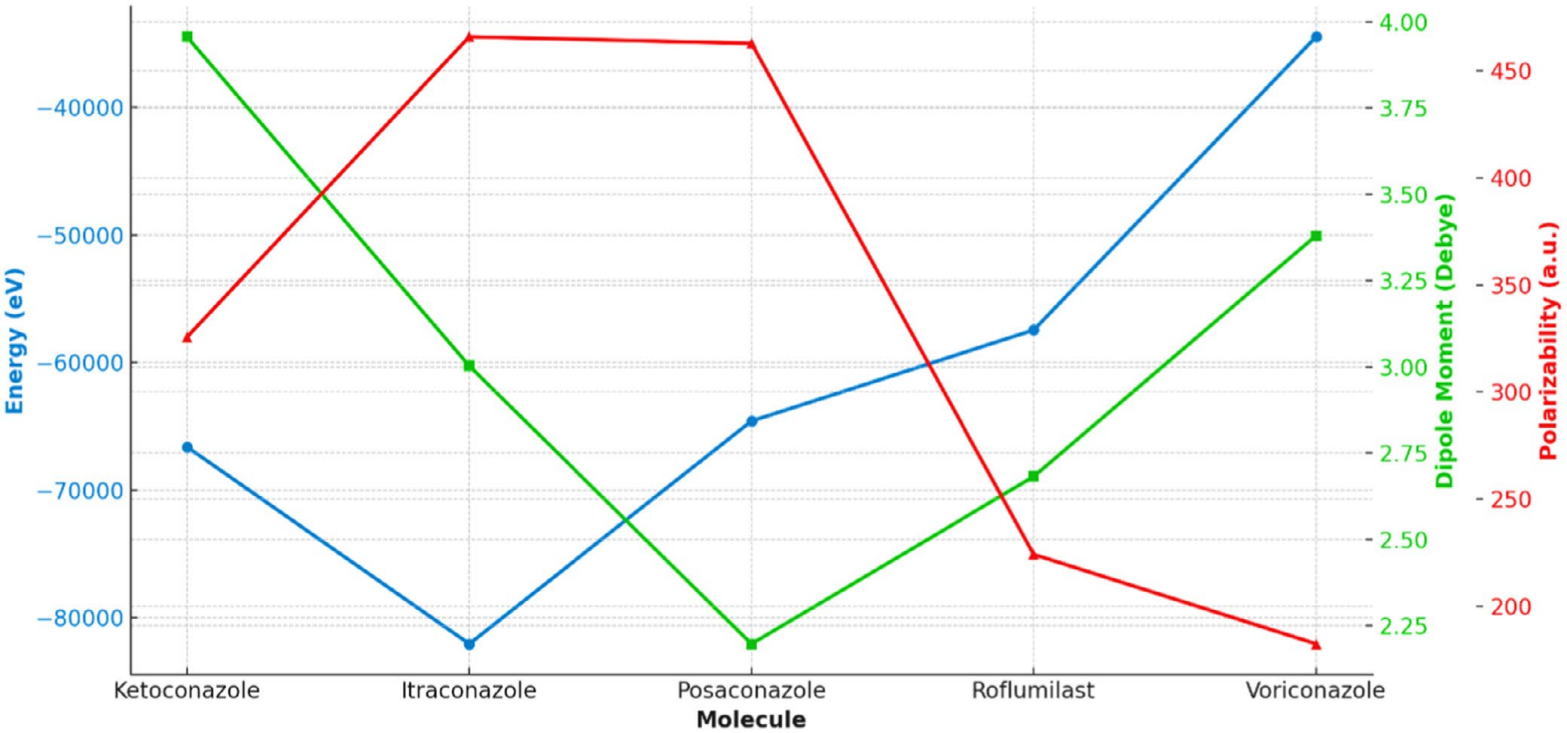
Comparative analysis of molecular properties: energy, dipole moment and polarizability of triazole derivatives and roflumilast.

Figure 9 illustrates a comparative analysis of five key molecules ketoconazole, itraconazole, posaconazole, roflumilast and voriconazole based on three physicochemical properties: total energy (eV), dipole moment (Debye) and polarizability (a.u.). The energy values, represented by the blue line, show that Itraconazole possesses the lowest energy, indicating greater thermodynamic stability, while Voriconazole exhibits the highest energy, suggesting lower stability. Dipole moment, shown in green, reflects the molecular polarity, with Ketoconazole having the highest dipole moment (~3.95 Debye), indicating strong polarity, which may affect solubility and intermolecular interactions. Conversely, Posaconazole has the lowest dipole moment, suggesting a more hydrophobic nature. The red curve represents polarizability, a measure of the molecule's electron cloud distortion under an electric field. Itraconazole and Posaconazole exhibit significantly higher polarizability values, implying greater electronic flexibility and potential for interaction with biological targets. In contrast, Voriconazole shows the lowest polarizability, correlating with reduced responsiveness to external fields. Overall, itraconazole demonstrates a unique combination of low energy, moderate dipole moment and high polarizability, making it a promising candidate for further pharmacological exploration, especially in drug repurposing strategies targeting inflammatory pathways such as PDE‐4 inhibition.

**FIGURE 10:**
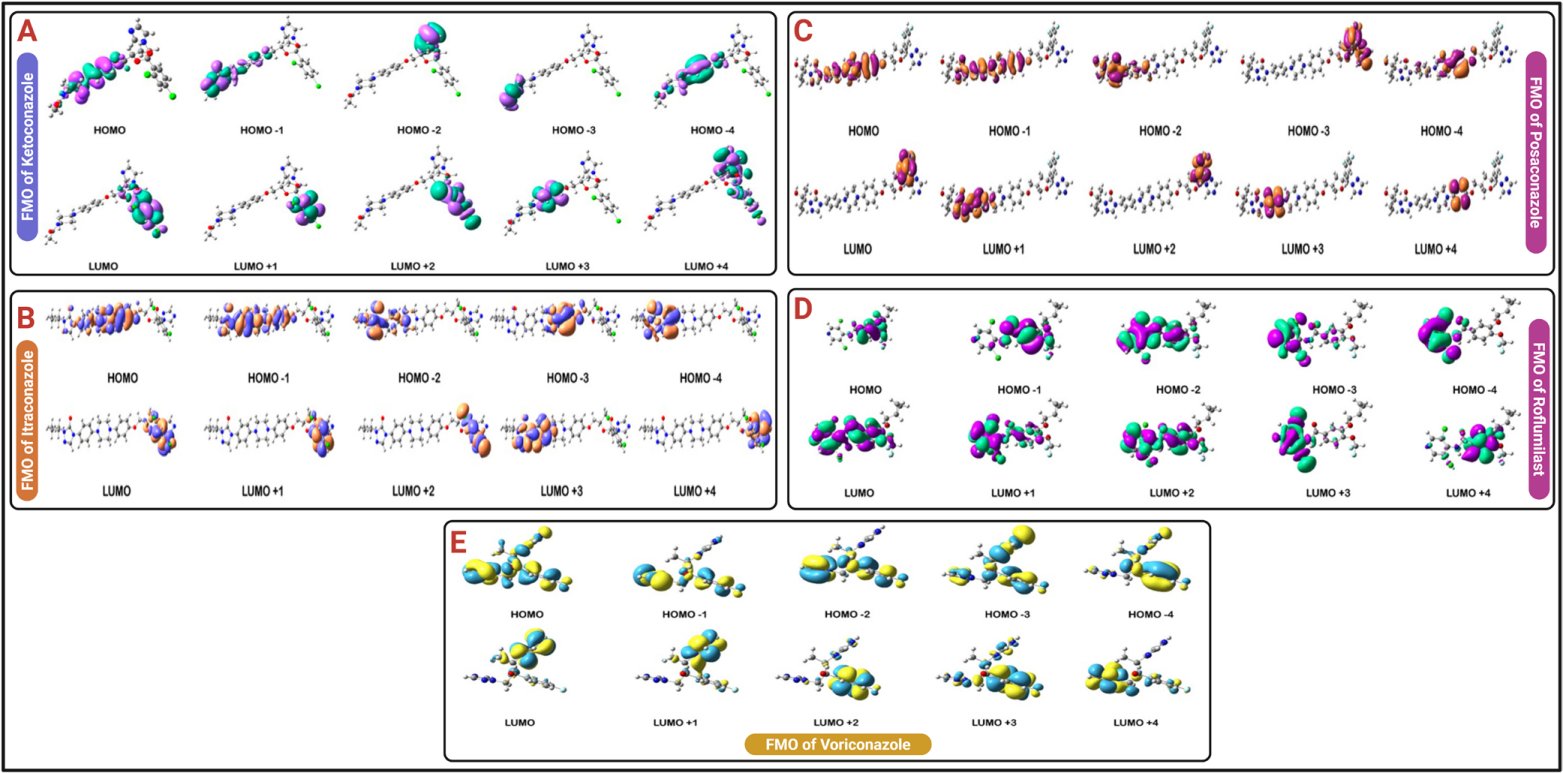
(a) FMO of ketoconazole; (b) FMO of itraconazole; (c) FMO of posaconazole; (d) FMO of roflumilast; (e) FMO of voriconazole.

**FIGURE 11:**
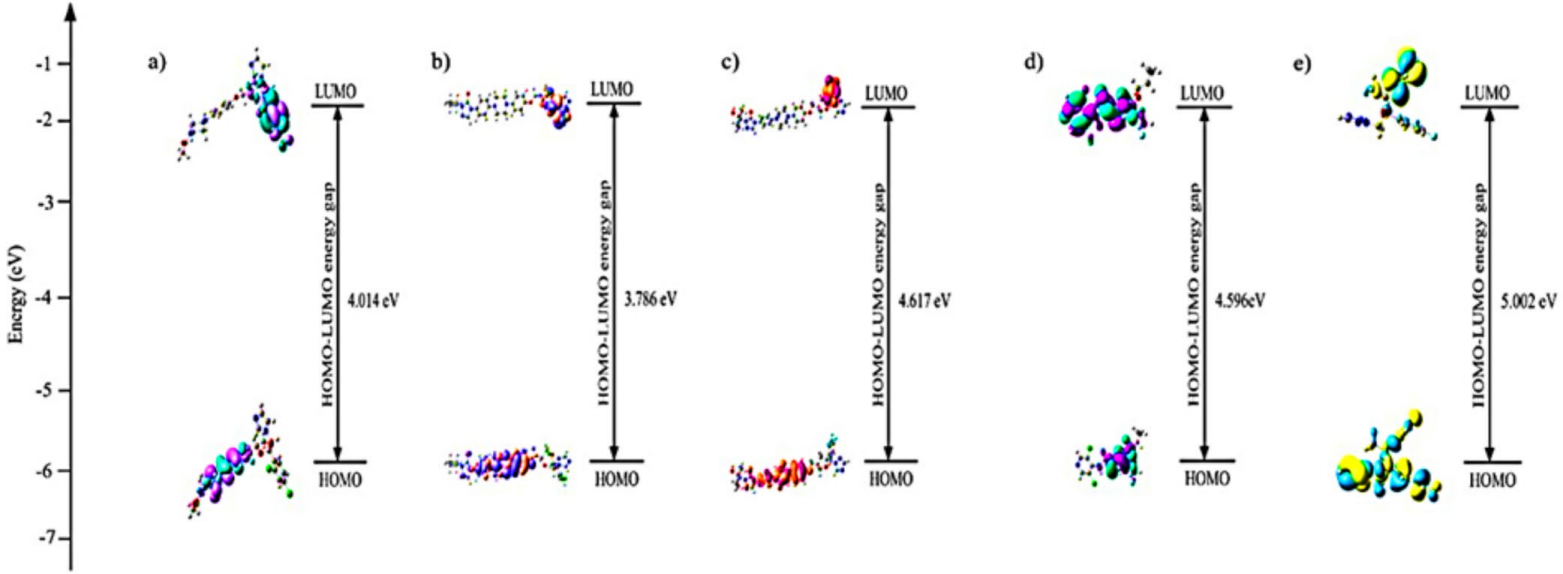
HOMO‐LUMO gap of: (a) ketoconazole, (b) itraconazole, (c) posaconazole, (d) roflumilast, (e) voriconazole.

The frontier molecular orbitals (FMOs) specifically the highest occupied molecular orbital (HOMO) and lowest unoccupied molecular orbital (LUMO) play a critical role in predicting chemical behaviour, including electron donation and acceptance. The energy gap (Δ*E = E*_LUMO_ *– E*_HOMO_) reflects molecular reactivity. Smaller ΔE values imply higher chemical reactivity and potential for intramolecular charge transfer (ICT). HOMO‐LUMO orbitals of all studied molecules are visualised in Figure 10, while the HOMO‐LUMO energy gaps are graphically summarised in Figure 11.

**FIGURE 12:**
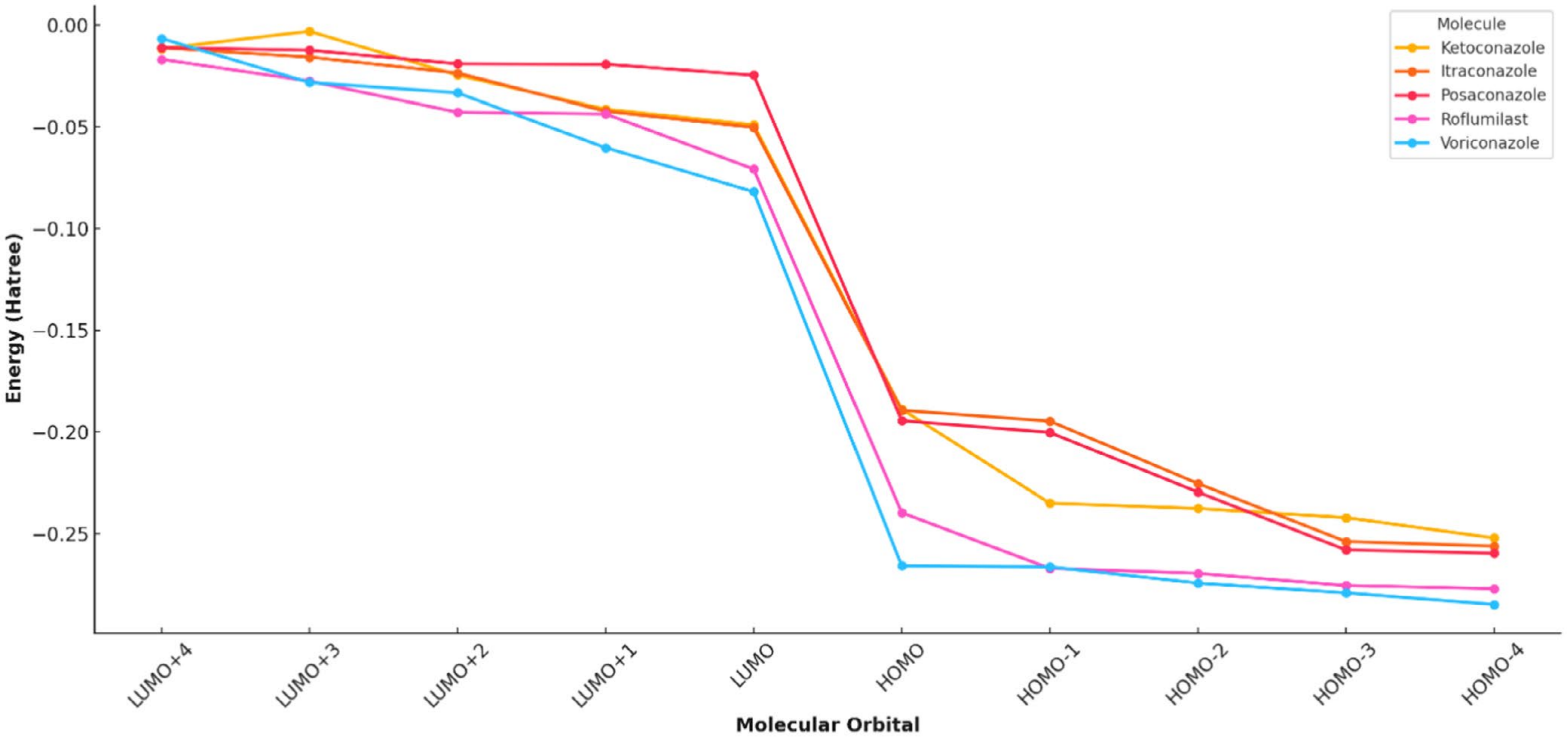
HOMO‐LUMO orbital energies of selected triazoles.

The frontier molecular orbital analysis, as presented in Figure 12 and Tables 3, 4, 5, 6, 7, offers critical insights into the electronic properties of the triazole antifungal agents and the PDE‐4 inhibitor Roflumilast. The HOMO (Highest Occupied Molecular Orbital) and LUMO (Lowest Unoccupied Molecular Orbital) energy levels dictate the molecule's electronic excitation behaviour, chemical reactivity and stability. Among all compounds, Voriconazole exhibited the most negative HOMO energy at −0.2657 Hatree and the lowest LUMO value at −0.0819 Hatree, indicating a large HOMO–LUMO energy gap. This suggests high kinetic stability and low chemical reactivity, aligning with its pharmacological durability. Conversely, ketoconazole showed a relatively less negative HOMO value (−0.1888 Hatree) and a LUMO energy of −0.0489 Hatree, implying a narrower energy gap and greater chemical reactivity, potentially enabling stronger interactions with biological targets.

**TABLE 3:** HOMO‐LUMO values of ketoconazole.

Serial number	Molecular orbital	Energy (Hatree)
144	LUMO+4	−0.0116
143	LUMO+3	−0.0031
142	LUMO+2	−0.0247
141	LUMO+1	−0.0413
140	LUMO	−0.0489
139	HOMO	−0.1888
138	HOMO−1	−0.2349
137	HOMO−2	−0.2375
136	HOMO−3	−0.2420
135	HOMO−4	−0.2520

**TABLE 4:** HOMO‐LUMO values of itraconazole.

Serial number	Molecular orbital	Energy (Hatree)
190	LUMO+4	−0.0112
189	LUMO+3	−0.0158
188	LUMO+2	−0.0236
187	LUMO+1	−0.0423
186	LUMO	−0.0502
185	HOMO	−0.01893
184	HOMO−1	−0.1946
183	HOMO−2	−0.2253
182	HOMO−3	−0.2537
181	HOMO−4	−0.2560

**TABLE 5:** HOMO‐LUMO values of posaconazole.

Serial number	Molecular orbital	Energy (Hatree)
190	LUMO+4	−0.0111
189	LUMO+3	−0.0124
188	LUMO+2	−0.0191
187	LUMO+1	−0.0193
186	LUMO	−0.0247
185	HOMO	−0.1944
184	HOMO−1	−0.2002
183	HOMO−2	−0.2295
182	HOMO−3	−0.2578
181	HOMO−4	−0.2595

Itraconazole and Posaconazole showed comparable electronic behaviours, with Itraconazole possessing a HOMO energy of −0.1893 Hatree and Posaconazole at −0.1944 Hatree. Both molecules have slightly more stabilised LUMOs than Ketoconazole, at −0.0502 and −0.0247 Hatree, respectively (Figure 12). Notably, Posaconazole displayed the smallest energy difference between its LUMO and LUMO+1 (−0.0247 to −0.0193 Hatree), indicating closely packed virtual orbitals that may facilitate rapid electron transitions under perturbation.

**TABLE 6:** HOMO‐LUMO values of roflumilast.

Serial number	Molecular orbital	Energy (Hatree)
108	LUMO+4	−0.0169
107	LUMO+3	−0.0276
106	LUMO+2	−0.0428
105	LUMO+1	−0.0436
104	LUMO	−0.0707
103	HOMO	−0.2396
102	HOMO−1	−0.2669
101	HOMO−2	−0.2694
100	HOMO−3	−0.2754
99	HOMO−4	−0.2769

Roflumilast, the non‐triazole PDE‐4 inhibitor, demonstrated a distinctly low HOMO energy at −0.2396 Hatree and a moderately stabilised LUMO at −0.0707 Hatree. This broader HOMO‐LUMO gap signifies its lower electrophilicity and possibly reduced likelihood of undergoing unintended off‐target redox reactions. However, the higher energy of its LUMO+4 (−0.0169 Hatree) in comparison to the antifungals suggests a potentially reduced capability to participate in charge‐transfer complexes beyond the ground‐state transitions.

The observed orbital energy trends are reflective of the molecular architecture and degree of π‐conjugation in the respective scaffolds. Compounds with lower HOMO values are more stable toward oxidative processes, while those with low‐lying LUMOs are more prone to accept electrons, indicative of enhanced electrophilic potential. From a drug design perspective, the HOMO‐LUMO gaps provide a theoretical basis for anticipating pharmacological activity, membrane permeability and potential redox behaviour in vivo. These findings are essential in rationalising the dual potential of triazole agents not only as antifungals but also as modulators of inflammatory pathways via interaction with targets such as PDE‐4.

Tables 3, 4, 5, 6, 7 detail the individual orbital energy levels for each compound. Among the triazoles, posaconazole presented the lowest LUMO energy (−0.67 eV), suggesting it can readily accept electrons, a feature that enhances its electrophilic behaviour. It also had a relatively low HOMO energy (−5.28 eV), resulting in a Δ*E* of 4.61 eV (Table 7). These values indicate a balance between stability and reactivity, essential for effective protein binding.

**TABLE 7:** HOMO‐LUMO values of voriconazole.

Serial number	Molecular orbital	Energy (Hatree)
95	LUMO+4	−0.0066
94	LUMO+3	−0.0283
93	LUMO+2	−0.0333
92	LUMO+1	−0.0602
91	LUMO	−0.0819
90	HOMO	−0.2657
89	HOMO−1	−0.2662
88	HOMO−2	−0.2742
87	HOMO−3	−0.2790
86	HOMO−4	−0.2847

**FIGURE 13:**
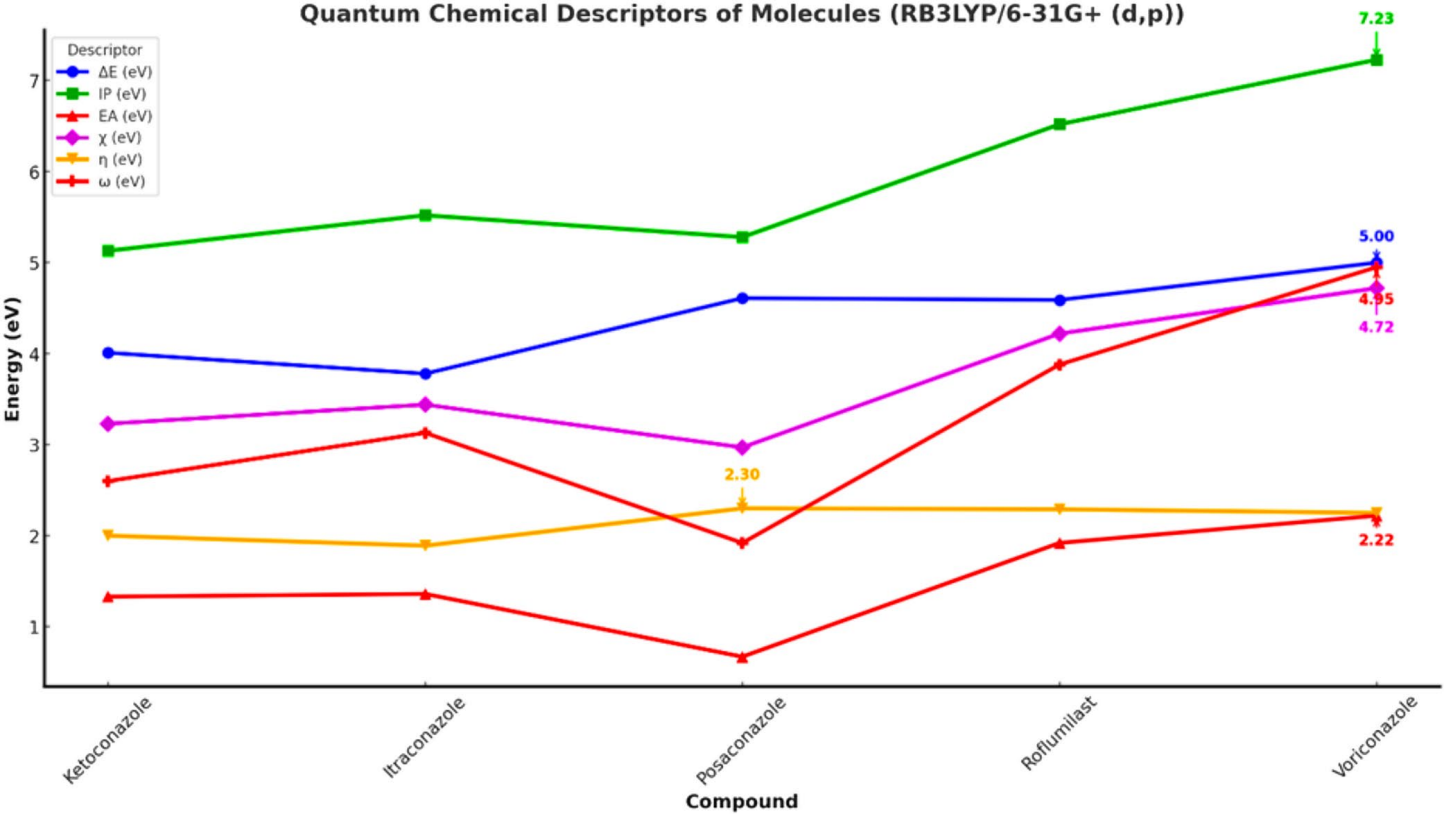
Quantum chemical descriptors of molecules.

The quantum chemical descriptors derived using the RB3LYP/6‐31G+(d,p) functional provide a comprehensive understanding of the electronic characteristics and reactivity profiles of the studied compounds. Among the five molecules, Voriconazole displayed the highest ionisation potential (IP) of 7.23 eV and electron affinity (EA) of 2.22 eV, indicating its strong resistance to oxidation and a significant tendency to accept electrons. This results in a high electronegativity (*χ* = 4.72 eV) and global electrophilicity index (*ω* = 4.95 eV), suggesting Voriconazole's pronounced electron‐accepting nature and potential biological reactivity, particularly relevant in target‐ligand interactions involving electrophilic centers. Despite its high reactivity, the molecule maintains a high chemical hardness (*η* = 2.25 eV), reflecting substantial kinetic stability and reduced polarizability. Roflumilast, a known PDE‐4 inhibitor, closely follows with an IP of 6.52 eV and EA of 1.92 eV, translating to a high electronegativity (*χ* = 4.22 eV) and hardness (*η* = 2.29 eV), as well as a significant electrophilicity (*ω* = 3.88 eV). This combination suggests Roflumilast's balanced profile of reactivity and stability, making it a favourable candidate for drug repurposing in inflammatory conditions like COVID‐19, where precise molecular targeting and moderate reactivity are desirable. In contrast, posaconazole exhibited the lowest electron affinity (0.67 eV) and electronegativity (2.97 eV), alongside the highest chemical hardness (*η* = 2.30 eV). These values suggest strong kinetic resistance to deformation and low electrophilic reactivity (*ω* = 1.92 eV), positioning posaconazole as a chemically stable, less reactive antifungal agent with potential advantages in minimising off‐target interactions and toxicity. However, its relatively wide HOMO‐LUMO gap (Δ*E* = 4.61 eV) reinforces its high intrinsic stability. Itraconazole and ketoconazole presented intermediate profiles. Itraconazole showed slightly higher IP and EA than ketoconazole (5.52 and 1.36 eV vs. 5.13 and 1.33 eV, respectively), and marginally higher electronegativity (3.44 vs. 3.23 eV), suggesting a more stable and reactive electronic framework. Interestingly, Ketoconazole had the highest softness (*σ* = 0.5) and lower electrophilicity (*ω* = 2.60 eV), indicating moderate reactivity with a potentially flexible electron distribution, possibly beneficial in binding adaptability within target active sites. The global hardness (*η*) and softness (*σ*) descriptors also provide insight into the susceptibility of each molecule to undergo electron redistribution. While posaconazole and roflumilast share comparable hardness values, Posaconazole's low electrophilicity contrasts sharply with Roflumilast's high ω, highlighting fundamental differences in their electronic behaviours despite structural similarities. These variations emphasise how subtle electronic differences can critically influence the pharmacological and biochemical responses of structurally related compounds. Overall, this descriptor‐based quantum chemical analysis substantiates the electronic diversity among the selected antifungal agents and roflumilast, justifying their distinct binding potentials, reactivities and stability profiles. These findings, when integrated with docking and MD results, can elucidate structure–activity relationships (SAR) and help prioritise candidates for dual‐function therapeutic development targeting both fungal infections and inflammatory pathologies (Figure 13).

**FIGURE 14:**
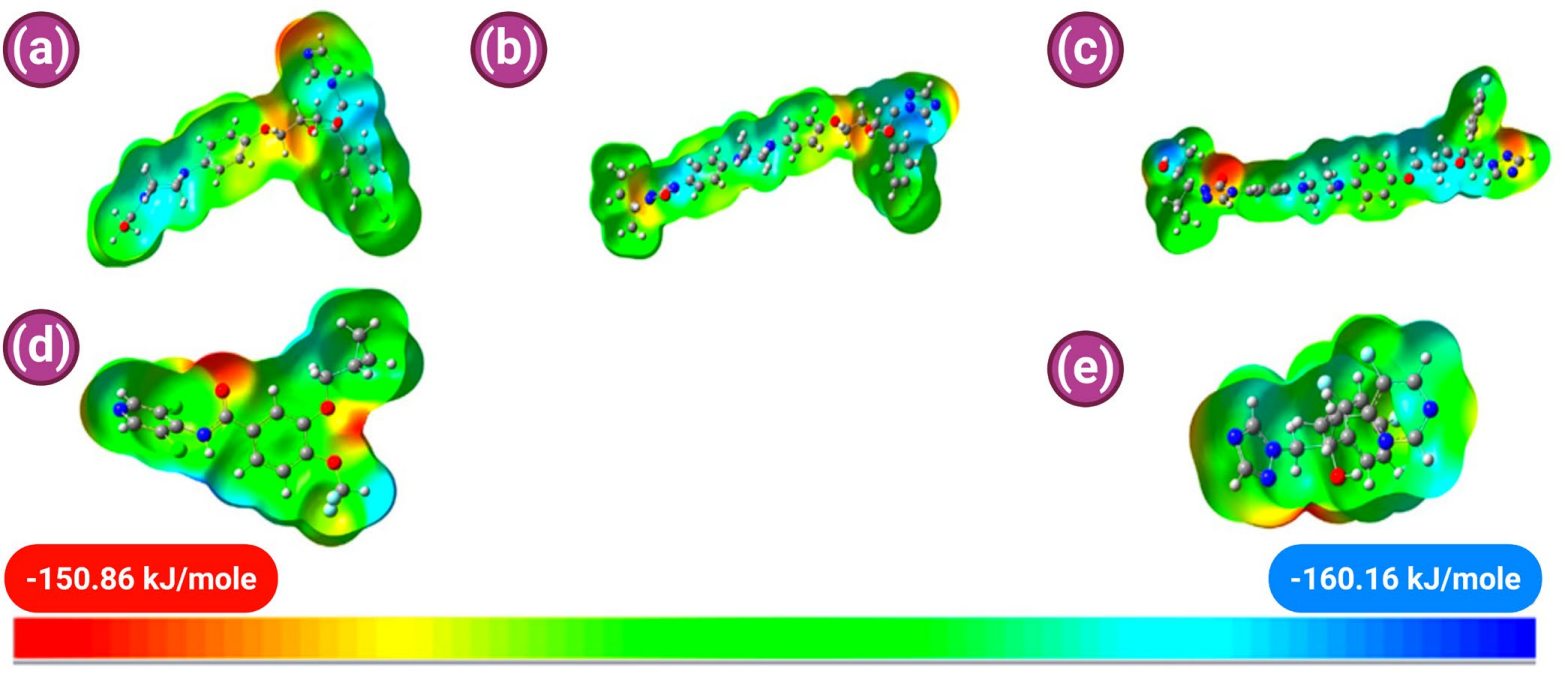
MEP of: (a) ketoconazole, (b) itraconazole, (c) posaconazole, (d) roflumilast, (e) voriconazole.

The molecular electrostatic potential (MEP) surfaces, calculated for each molecule and shown in Figure 14, provide insight into regions of electrophilic and nucleophilic attack. Colour gradients from red (electron‐rich) to blue (electron‐poor) highlight key reactive sites. In all triazoles, high electronegativity was localised around oxygen atoms, particularly those in hydroxyl or ether groups, suggesting a strong potential for hydrogen bonding with PDE‐4 residues.

**FIGURE 15:**
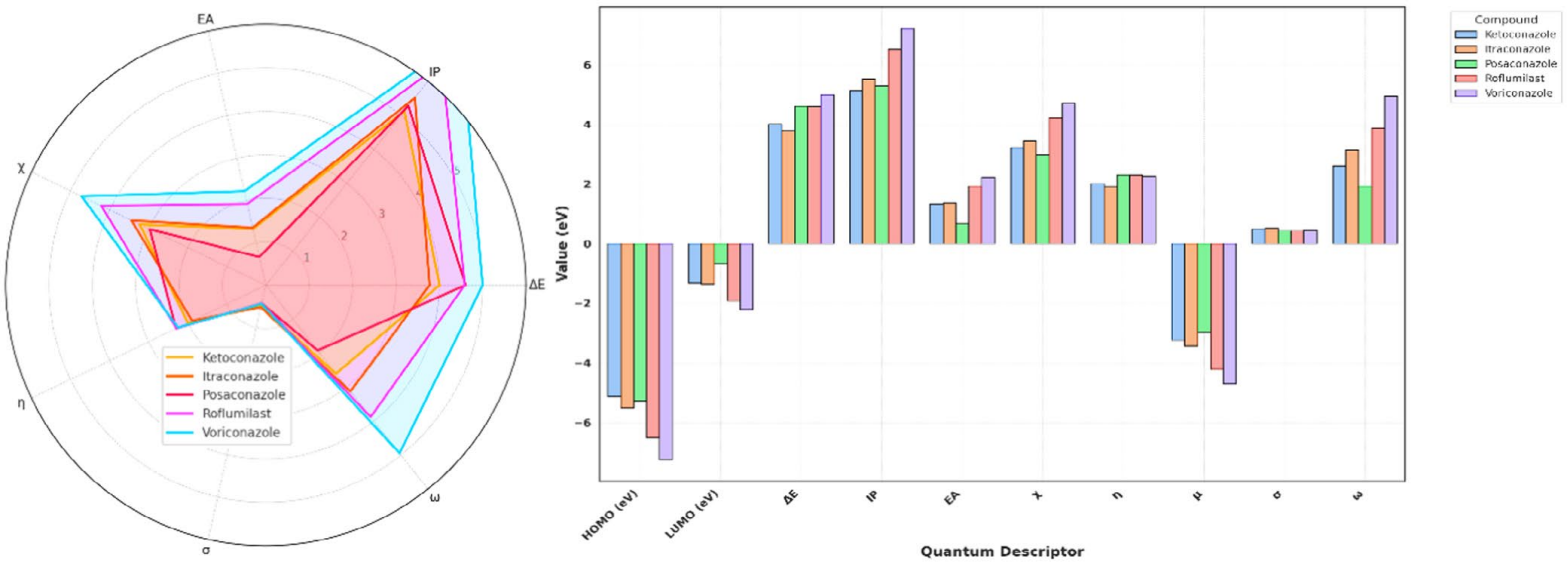
Comparison of quantum descriptors across compounds.

The quantum chemical parameters computed for ketoconazole, itraconazole, posaconazole and the reference PDE‐4 inhibitor roflumilast using the RB3LYP/6‐31G+(d,p) level of theory provide valuable insight into the electronic reactivity and stability profiles of these molecules. Among the triazoles, itraconazole exhibits the lowest HOMO energy (−5.52 eV), indicating the strongest electron‐donating resistance, while posaconazole has the highest LUMO energy (−0.67 eV), suggesting a higher tendency to accept electrons. The energy gap (ΔE), an indicator of molecular stability and reactivity, is narrowest for itraconazole (3.78 eV) and widest for posaconazole (4.61 eV). A smaller ΔE denotes higher chemical reactivity and softer character, thus suggesting itraconazole may interact more efficiently with biological targets. Ketoconazole and itraconazole share comparable ionisation potentials (IP = 5.13 and 5.52 eV, respectively), but differ in electron affinities (EA), with itraconazole having a higher EA (1.36 eV), implying a greater ability to accommodate an additional electron. Posaconazole shows the lowest EA (0.67 eV), correlating with its high chemical hardness (*η* = 2.30 eV), and hence, lower polarizability. Electronegativity (*χ*) and electrophilicity index (*ω*) are also highest for itraconazole (*χ* = 3.44 eV, *ω* = 3.13), suggesting strong electron‐accepting power, possibly enhancing its pharmacodynamic interaction with PDE‐4. Furthermore, the softness parameter (*σ*), which is the reciprocal of hardness, places itraconazole (*σ* = 0.52) as a relatively softer molecule compared to posaconazole (*σ* = 0.43), reinforcing its potential to participate in biological electron transfer. The chemical potential (*μ*) and Mulliken electronegativity trend (inversely correlated) also support these findings, with itraconazole and ketoconazole showing more negative *μ* values, signifying higher electron richness. Comparatively, roflumilast serves as a standard with balanced electronic characteristics, but itraconazole stands out for its optimal reactivity, electrophilicity and moderate Δ*E* indicating a strong potential for PDE‐4 inhibition via stable binding and favourable frontier orbital alignment. Among the molecules, posaconazole displayed a high electrophilicity index (*ω* = 1.92), a lower hardness (*η* = 2.30) and moderate electronegativity (*χ* = 2.97), suggesting a soft, reactive and electrophilic nature conducive for enzyme inhibition (Table 8 and Figure 15).

**TABLE 8:** Quantum descriptors for molecule 1 in electron volts (eV) with the RB3LYP‐631G+ (d,p) functional.

Compound	HOMO (eV)	LUMO (eV)	Δ*E*	IP	EA	*χ*	*η*	*μ*	*σ*	*ω*
Ketoconazole	−5.13	−1.33	4.01	5.13	1.33	3.23	2.00	−3.23	0.50	2.60
Itraconazole	−5.52	−1.36	3.78	5.52	1.36	3.44	1.89	−3.44	0.52	3.13
Posaconazole	−5.28	−0.67	4.61	5.28	0.67	2.97	2.30	−2.97	0.43	1.92
Roflumilast	−6.52	−1.92	4.59	6.52	1.92	4.22	2.29	−4.22	0.43	3.88
Voriconazole	−7.23	−2.22	5.00	7.23	2.22	4.72	2.25	−4.72	0.44	4.9

In comparison, roflumilast exhibited a slightly lower Δ*E* (4.59 eV) and a significantly higher electrophilicity index (*ω* = 3.88), making it a robust reference molecule. Nevertheless, posaconazole's favourable HOMO‐LUMO alignment and MEP profile support its potential as a bioisosteric candidate for PDE‐4 inhibition. This quantum mechanical analysis, when coupled with docking and simulation data, underscores posaconazole's aptitude not only for binding affinity but also for dynamic adaptability within the PDE‐4 active site. Such findings justify its selection for further in vitro and in vivo studies focused on cytokine storm attenuation through targeted PDE‐4 inhibition.

### 3.5 Docking Revalidation and Cluster Analysis

**FIGURE 16:**
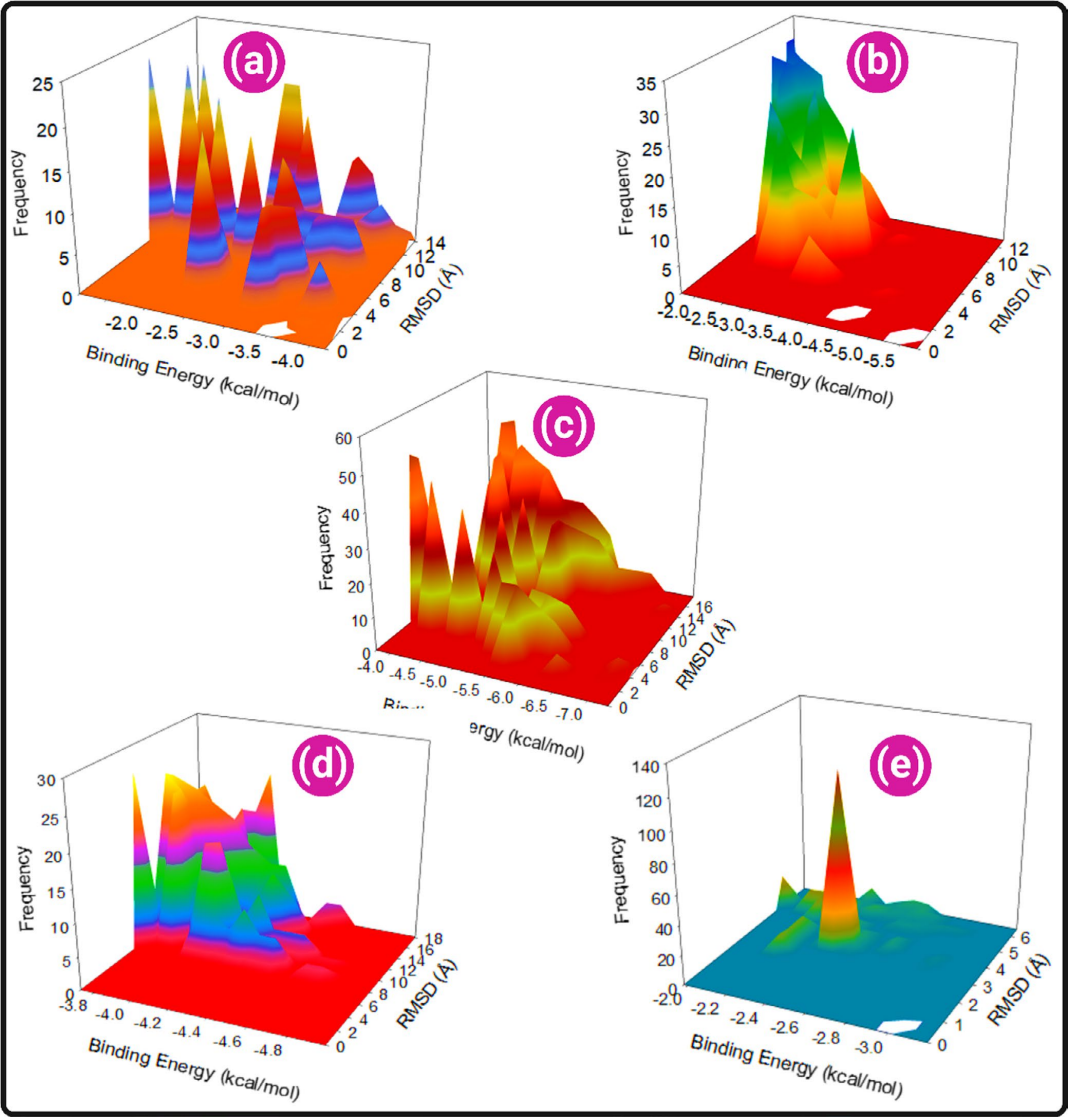
(a–e) 3D RMSD versus binding energy cluster map of 50 poses of each ligand (roflumilast, a standard PDE4 inhibitor; posaconazole, ketoconazole, itraconazole and voriconazole) docked with PDE4 protein exhibiting the frequency of populations at 2.0 tolerance level.

Docking was revalidated using AutoDock 4.2.6. Cluster analysis (RMSD = 0.0 Å) revealed binding population frequencies. The cluster analysis exhibited the frequency of numbers of populations in each cluster (Figure 16).

**FIGURE 17:**
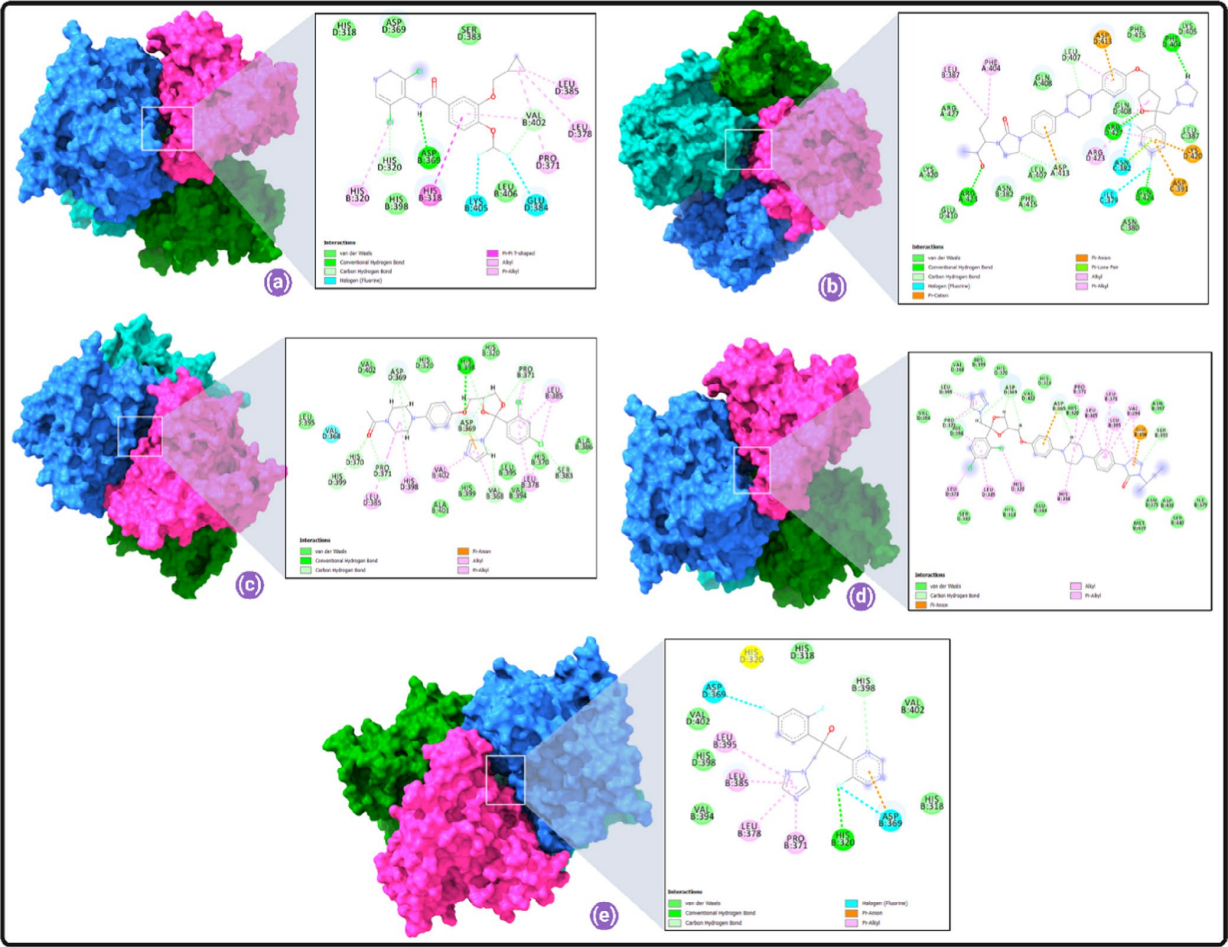
(a) Surface 3D view of PDE4 exhibiting a deep core of the binding pocket accommodating the ligand roflumilast; and 2D interactions of the roflumilast binding pocket in the PDE‐4 protein; (b) Surface 3D view of PDE4 exhibiting a deep core of the binding pocket accommodating the ligand posaconazole; and 2D interactions of the posaconazole binding pocket in the PDE‐4 protein; (c) Surface 3D view of PDE4 exhibiting a deep core of the binding pocket accommodating the ligand ketoconazole; and 2D interactions of the ketoconazole binding pocket in the PDE‐4 protein; (d) Surface 3D view of PDE4 exhibiting a deep core of the binding pocket accommodating the ligand itraconazole; and 2D interactions of the itraconazole binding pocket in the PDE‐4 protein; (e) Surface 3D view of PDE4 exhibiting a deep core of the binding pocket accommodating the ligand voriconazole; and 2D interactions of the voriconazole binding pocket in the PDE‐4 protein.

The lowest binding energy for PDE‐4‐roflumilast was −4.95 kcal/mol (Figure 17a). For PDE‐4‐posaconazole, binding was stronger with −7.29 kcal/mol (Figure 17b). Ketoconazole and itraconazole followed with −5.76 kcal/mol (Figure 17c) and −4.17 kcal/mol (Figure 17d), respectively. Voriconazole showed the weakest interaction (−3.18 kcal/mol) (Figure 17e).

The residue‐level interactions for each ligand were extensively documented. Posaconazole formed conventional hydrogen bonds with ARG 423, ARG 427, PHE 404 and GLN 424, and non‐covalent interactions such as π‐anion, π‐cation, halogen and van der Waals bonds, indicating robust complex stability (Figure 17b).

### 3.6 MD Simulation

**FIGURE 18:**
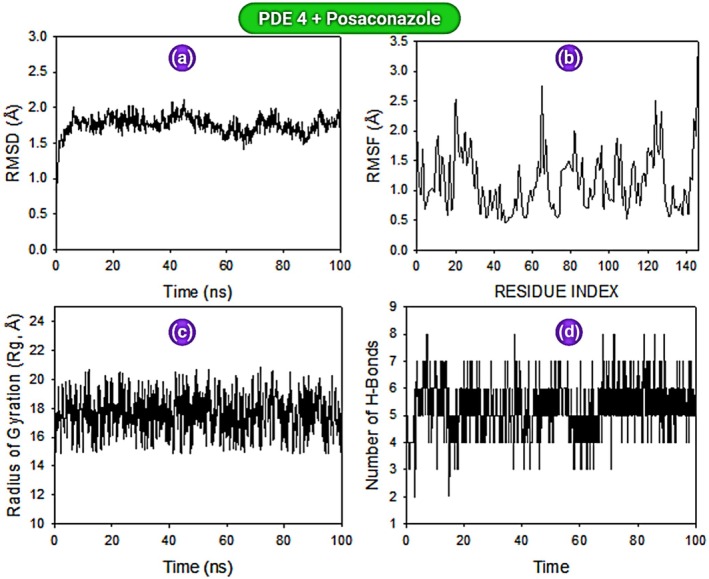
MD simulation analysis of 100 ns trajectories of: (a) Cα backbone RMSD of PDE4‐posaconazole, (b) RMSF of Cα backbone of PDE4‐posaconazole, (c) Cα backbone radius of gyration (Rg) of PDE4‐posaconazole, (d) formation of hydrogen bonds in PDE4‐Posaconazole.

**FIGURE 19:**
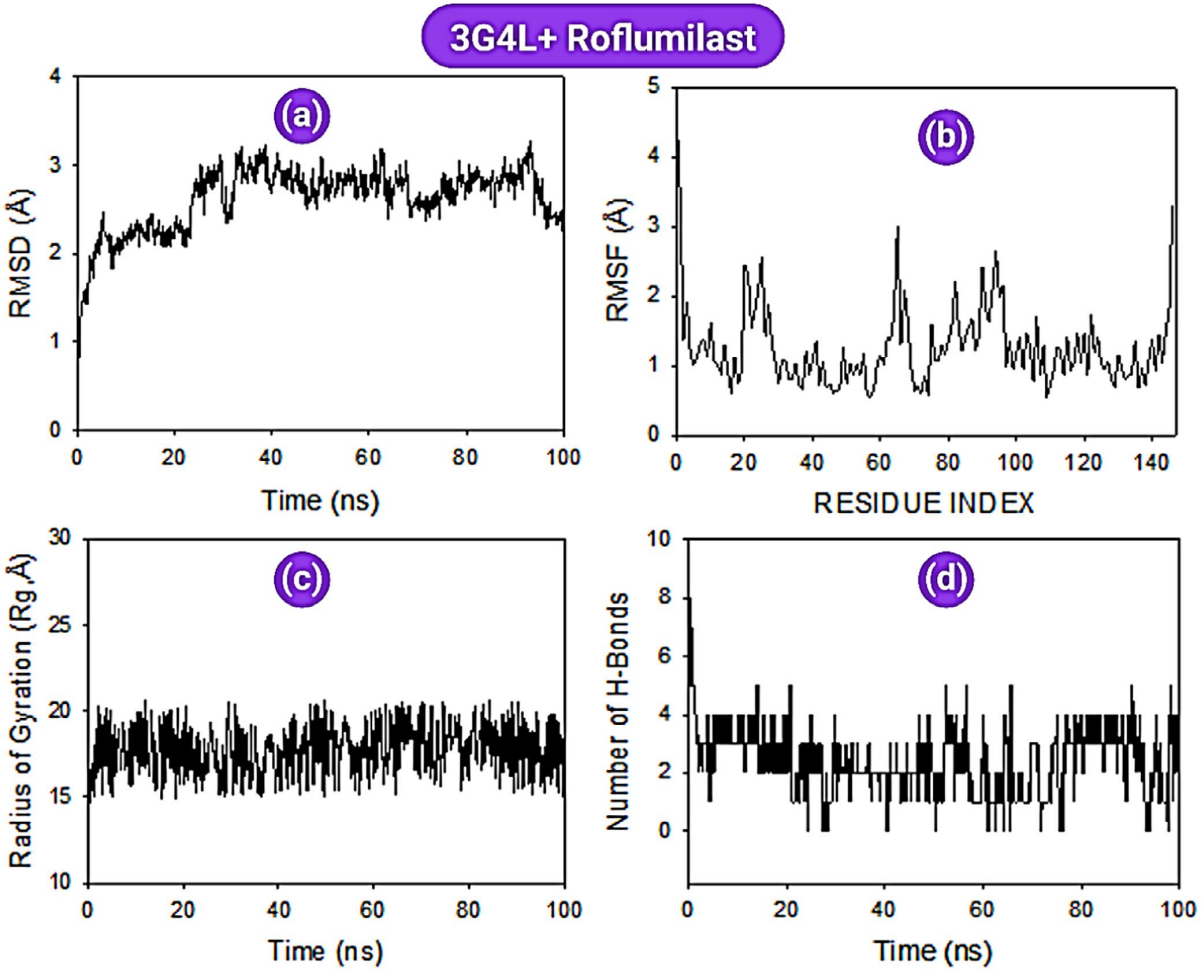
MD simulation analysis of 100 ns trajectories of: (a) Cα backbone RMSD of 3G4L + roflumilast, (b) RMSF of Cα backbone of 3G4L + Roflumilast, (c) Cα backbone radius of gyration (Rg) of 3G4L + roflumilast, (d) formation of hydrogen bonds in 3G4L + roflumilast.

MD simulations (100 ns) were conducted for PDE‐4 + roflumilast and PDE‐4 + posaconazole complexes. Root Mean Square Deviation (RMSD): RMSD of the Cα‐backbone in the PDE‐4 + posaconazole complex remained within a narrow range (2.0–2.4 Å) for the entire 100 ns, indicating strong conformational stability (Figure 18a). In contrast, the PDE‐4 + roflumilast complex exhibited significant fluctuations up to 40 ns, reaching 2.8 Å, before stabilising (Figure 19a). The late‐stage drop in RMSD in the roflumilast complex suggests a conformational adjustment possibly due to reduced binding affinity. *Root Mean Square Fluctuation (RMSF)*: RMSF analysis indicated localised flexibility in a few loop regions. For posaconazole, noticeable spikes were observed at residues 19, 64, and 124–127, corresponding to non‐structured loop areas (Figure 18b). These fluctuations, however, remained below 3 Å. The roflumilast complex showed minor spikes at residues 20–24, 65 and 90–94, again localised and moderate in magnitude (Figure 19b). *Radius of Gyration (Rg)*: Both complexes maintained a consistent radius of gyration throughout the simulation (average ~22.4 Å), indicating no global unfolding or compactness loss during simulation. This implies that the protein retained its tertiary structure integrity in both ligand‐bound states (Figures 18c and 19c). *Hydrogen Bond Analysis*: Hydrogen bonding analysis revealed the PDE‐4 + roflumilast complex had an average of 5 hydrogen bonds sustained over the 100 ns period (Figure 19d), suggesting a stable interaction network. PDE‐4 + posaconazole maintained an average of 2 hydrogen bonds throughout the simulation (Figure 18d). Although fewer, these bonds were strong and persistent, further validated by low RMSD values.

### 3.7 MM‐GBSA Binding Free Energy Calculation

**FIGURE 20:**
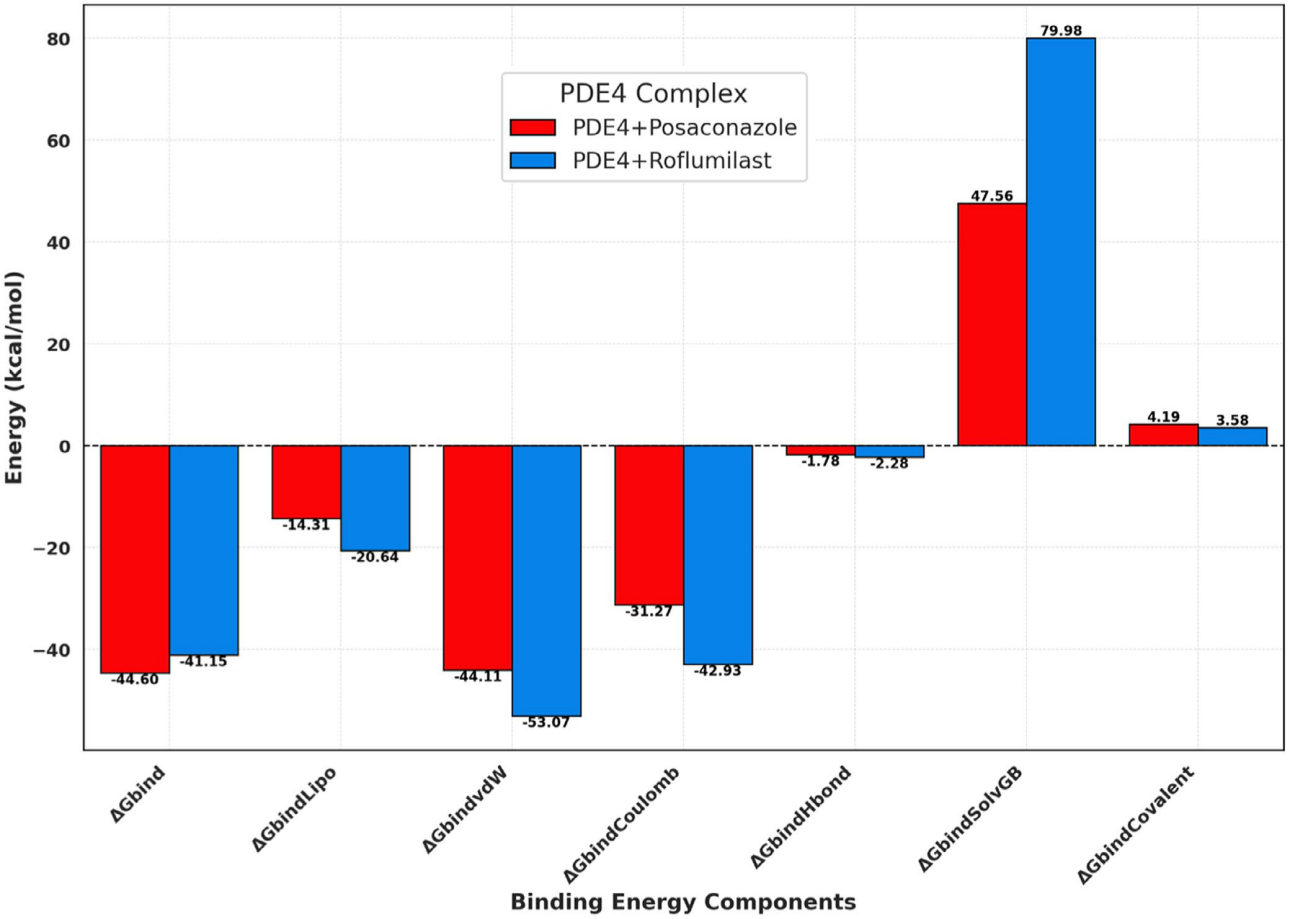
Component‐wise binding energy analysis of PDE4 complexes.

Binding free energy and energetic contributions were computed using MM‐GBSA. Figure 20 and Table 9 present the breakdown of each energy component. Posaconazole showed a Δ*G*_bind_ of −44.60 kcal/mol, while roflumilast exhibited −41.15 kcal/mol. Major contributors to complex stability included:

- Δ*G*_bind_Coulomb (electrostatic interaction)
- Δ*G*_bind_vdW (van der Waals forces)
- Δ*G*_bind_Lipo (lipophilic interactions)

**TABLE 9:** Binding free energy components for the PDE‐4 complex calculated by MM‐GBSA.

Energies (kcal/mol)	PDE4 + Posaconazole	PDE4 + Roflumilast
Δ*G*_bind_	−44.60	−41.15
Δ*G*_bind_Lipo	−14.31	−20.64
Δ*G*_bind_vdW	−44.11	−53.07
Δ*G*_bind_Coulomb	−31.27	−42.93
Δ*G*_bind_H_bond_	−1.78	−2.28
Δ*G*_bind_SolvGB	47.56	79.98
Δ*G*_bind_Covalent	4.19	3.58

In contrast, Δ*G*_bind_SolvGB and Δ*G*_bind_Covalent had destabilising effects. The lower total binding energy for posaconazole supports its strong affinity and further justifies its selection for potential PDE‐4 inhibition.

According to computational calculations outlined in Table 10, posaconazole promises to be a leading candidate for PDE‐4 inhibitors among the triazoles since it demonstrates the best‐predicted binding affinity (−7.29 kcal/mol), which is much less than a reference drug, roflumilast (−4.95 kcal/mol), as well as a reasonable electronic profile featuring a moderate HOMO‐LUMO gap (4.61 eV) that indicates its stability, low electrophilicity (*ω* = 1.92 eV) that points to reduced nonspecific reactivity and high chemical hardness (*η* = 2.30 eV) signifying resistance to electron transfer deformation. Although itraconazole had the smallest HOMO‐LUMO gap (3.78 eV) and largest electrophilicity (*ω* = 3.13 eV), which characterises high intrinsic chemical reactivity, its smaller binding energy (−4.17 kcal/mol) places it behind posaconazole. Ketoconazole had moderate binding (−5.76 kcal/mol) and reactivity, whereas voriconazole had the weakest binding (−3.18 kcal/mol) and lowest reactivity (largest Δ*E* = 5.00 eV, highest *ω* = 4.90 eV), which makes it the least appropriate as a PDE‐4 inhibitor. In this integrated assessment approach, the high affinity and specificity of the interaction of posaconazole with the PDE‐4 catalytic site stand out as potentially great.

**TABLE 10:** Integrated computational comparison of PDE‐4 binding affinity, conformational stability and quantum chemical reactivity descriptors for triazole antifungals and roflumilast.

Compound	Docking binding energy (kcal/mol)	HOMO‐LUMO Gap (Δ*E*, eV)	Electrophilicity index (*ω*, eV)	Chemical hardness (*η*, eV)
Roflumilast (Reference)	−4.95	4.59	3.88	2.29
Posaconazole	−7.29	4.61	1.92	2.30
Ketoconazole	−5.76	4.01	2.60	2.00
Itraconazole	−4.17	3.78	3.13	1.89
Voriconazole	−3.18	5.00	4.90	2.25

## 4 Discussion

The outcomes of this study present a compelling case for the strategic repositioning of triazole antifungals as inhibitors of PDE‐4, highlighting their dual functionality in combating COVID‐19‐induced cytokine storms and fungal co‐infections. The molecular docking results demonstrated that posaconazole, ketoconazole and itraconazole exhibited higher binding affinities to PDE‐4 compared to the benchmark inhibitor, roflumilast. Among these, posaconazole was identified as the most promising candidate, with docking scores and molecular interactions that closely mimicked those of roflumilast, particularly involving critical residues such as THR 499, GLN 535, ILE 502, PHE 538 and MET 523. This structural convergence underscores the feasibility of these antifungals in targeting mammalian PDE‐4.

The quantum chemical analysis reinforced the binding potential observed in docking studies. Posaconazole's low HOMO‐LUMO energy gap, high electron affinity and elevated electrophilicity index reflected its strong reactivity and interaction capability with PDE‐4. The MEP further localised the reactive zones conducive for hydrogen bonding and nucleophilic attack, primarily near electronegative oxygen atoms. Importantly, the MD simulations offered valuable insights into the temporal stability of these complexes. The PDE‐4 + posaconazole complex displayed consistent RMSD values (2.0–2.4 Å), indicating stable conformational retention throughout the 100 ns trajectory. In contrast, PDE‐4 + roflumilast exhibited transient deviations and a notable drop in the final simulation phase, suggesting potential conformational instability. RMSF analysis revealed minimal backbone fluctuations for both complexes, although posaconazole exhibited slightly higher residue‐level flexibility in specific loop regions, which may contribute to its adaptive binding. Rg plots for both complexes confirmed the structural compactness of the protein throughout the simulations, indicating no unfolding or major destabilisation in ligand‐bound states. Notably, posaconazole maintained fewer but more consistent hydrogen bonds over time, signifying deep pocket integration and a sustained binding network—qualities critical for long‐term inhibition. MM‐GBSA binding free energy calculations further validated these findings, with posaconazole showing the most favourable total binding energy (−44.60 kcal/mol), primarily driven by electrostatic, van der Waals and lipophilic interactions. These results collectively point to a novel mechanism of action for triazoles beyond their antifungal role. By directly engaging the catalytic pocket of PDE‐4 and mirroring the interactions of established inhibitors, these agents demonstrate potential for immunomodulation via suppression of pro‐inflammatory cytokines. Given the overlapping prevalence of COVID‐19 and secondary fungal infections, such as mucormycosis, these dual‐function drugs may provide an efficient and targeted therapeutic approach. This is especially relevant during pandemics, where time, cost and drug availability are critical constraints.

Our study, therefore, offers a forward‐looking paradigm where antifungal triazoles could be reclassified as multi‐target agents capable of modulating host inflammatory pathways while treating opportunistic infections. The novelty of this investigation lies not only in the repurposing framework but also in the identification of posaconazole as a high‐affinity PDE‐4 binder. Its binding energetics, structural compatibility and dynamic stability establish a strong rationale for in vitro and in vivo validation. Future directions should include biochemical assays and pharmacodynamic evaluations to determine dose‐dependent PDE‐4 inhibition and subsequent cytokine suppression. If validated, this dual‐role strategy may revolutionise the management of inflammatory complications in viral infections, introducing a new therapeutic utility for a well‐characterised class of antifungal drugs.

## 5 Conclusion

This study presents a novel and scientifically grounded strategy for repurposing triazole antifungals as dual‐function agents capable of targeting both pathogenic fungi and the inflammatory cascades associated with severe COVID‐19. Through an integrative computational approach—including molecular docking, MD simulations, MM‐GBSA free energy calculations and DFT analyses—we identified posaconazole as a leading candidate with robust binding to the catalytic domain of PDE‐4. Its interaction profile paralleled that of roflumilast, a known PDE‐4 inhibitor, and was further validated by superior thermodynamic stability and structural adaptability under dynamic physiological conditions. Quantum chemical descriptor analysis revealed posaconazole's optimal balance between chemical stability and electrophilic reactivity, supported by a favourable HOMO‐LUMO energy gap and consistent MEP zones conducive to hydrogen bonding. These features collectively suggest that posaconazole can act not only as a potent antifungal but also as a modulator of cytokine‐mediated inflammation. Such dual‐action capability holds great promise in clinical settings, especially during pandemics where co‐infections and immune dysregulation occur simultaneously. The ability to inhibit PDE‐4 while retaining antifungal efficacy provides a strategic advantage in managing complex pathologies like COVID‐19–associated mucormycosis. The repositioning of triazoles—particularly posaconazole—represents a forward‐looking therapeutic concept that bridges infectious disease management and host‐directed immunomodulation. Future in vitro and in vivo investigations are essential to validate these findings, but the current evidence lays a strong foundation for translating this computational insight into clinical innovation.

## Author Contributions

**Hailah M. Almohaimeed:** conceptualization (equal), formal analysis (equal), investigation (equal), writing – original draft (equal). **Aniruddha Chatterjee:** data curation (equal), formal analysis (equal), investigation (equal), writing – original draft (equal). **Majedah Ramadan Alaqabawi:** methodology (equal), software (equal), validation (equal), writing – review and editing (equal). **Ayman Jafer:** data curation (equal), software (equal), validation (equal), writing – review and editing (equal). **Ahmed M. Basri:** conceptualization (equal), formal analysis (equal), validation (equal), writing – review and editing (equal). **Fayez Alsulaimani:** conceptualization (equal), investigation (equal), methodology (equal), writing – review and editing (equal). **Abdullah F. Shater:** conceptualization (equal), formal analysis (equal), writing – original draft (equal), writing – review and editing (equal). **Fayez M. Saleh:** data curation (equal), validation (equal), writing – original draft (equal), writing – review and editing (equal). **Bikram Dhara:** conceptualization (equal), software (equal), supervision (equal), validation (equal), writing – review and editing (equal). **Daniel Ejim Uti:** conceptualization (equal), formal analysis (equal), validation (equal), writing – review and editing (equal). **Esther Ugo Alum:** conceptualization (equal), validation (equal), writing – original draft (equal), writing – review and editing (equal).

## Ethics Statement

The authors have nothing to report.

## Consent

The authors have nothing to report.

## Conflicts of Interest

The authors declare no conflicts of interest.

## Data Availability

All used data is within the manuscript.
